# Comparative phyloproteomics identifies conserved plasmodesmal proteins

**DOI:** 10.1093/jxb/erad022

**Published:** 2023-01-14

**Authors:** Matthew G Johnston, Andrew Breakspear, Sebastian Samwald, Dan Zhang, Diana Papp, Christine Faulkner, Jeroen de Keijzer

**Affiliations:** Department of Cell and Developmental Biology, John Innes Centre, UK; Department of Cell and Developmental Biology, John Innes Centre, UK; Department of Cell and Developmental Biology, John Innes Centre, UK; Department of Cell and Developmental Biology, John Innes Centre, UK; Department of Cell and Developmental Biology, John Innes Centre, UK; Department of Cell and Developmental Biology, John Innes Centre, UK; Laboratory of Cell Biology, Wageningen University and Research, Wageningen, The Netherlands; Bielefeld University, Germany

**Keywords:** Arabidopsis, cell to cell communication, phyloproteomics, *Physcomitrium patens*, plasmodesmata, proteomics

## Abstract

Plasmodesmata are cytosolic bridges, lined by the plasma membrane and traversed by endoplasmic reticulum; plasmodesmata connect cells and tissues, and are critical for many aspects of plant biology. While plasmodesmata are notoriously difficult to extract, tissue fractionation and proteomic analyses can yield valuable knowledge of their composition. Here we have generated two novel proteomes to expand tissue and taxonomic representation of plasmodesmata: one from mature Arabidopsis leaves and one from the moss *Physcomitrium patens*, and leveraged these and existing data to perform a comparative analysis to identify evolutionarily conserved protein families that are associated with plasmodesmata. Thus, we identified β-1,3-glucanases, C2 lipid-binding proteins, and tetraspanins as core plasmodesmal components that probably serve as essential structural or functional components. Our approach has not only identified elements of a conserved plasmodesmal proteome, but also demonstrated the added power offered by comparative analysis for recalcitrant samples. Conserved plasmodesmal proteins establish a basis upon which ancient plasmodesmal function can be further investigated to determine the essential roles these structures play in multicellular organism physiology in the green lineages.

## Teaser

Plasmodesmata enable cell to cell communication for growth, development, and responses in plants. We used comparative analysis of plasmodesmal proteomes from divergent plants to identify conserved plasmodesmal elements.

## Introduction

Plasmodesmata are membrane-lined connections that traverse the cell wall and interconnect the cytoplasm, plasma membrane, and endoplasmic reticulum (ER) between plant cells. The direct cytosol–cytosol contact enables the sharing of resources and information, underpinning growth, developmental, and response processes (Benitez-Alfonso, 2014; Sevilem *et al.*, 2015; Brunkard and Zambryski, 2017; Cheval and Faulkner, 2018). Plasmodesmata are dynamic, responding to internal and external cues to create transient domains of connectivity within tissues. While it is established that their responses to a range of environmental signals are enabled by specialized signalling machinery (Cui and Lee, 2016; Cheval *et al.*, 2020), the molecular machinery that brings about their biogenesis and structure is less well defined.

To obtain a comprehensive overview of proteins present at plasmodesmata, and ultimately build understanding of their role in physiology and development, proteomic characterization of plasmodesmata-enriched fractions has been performed on multiple occasions (Faulkner *et al.*, 2005; Fernandez-Calvino *et al.*, 2011; Park *et al.*, 2017; Leijon *et al.*, 2018; Brault *et al.*, 2019). Such proteomes have provided valuable insights into plasmodesmal structure and function, identifying novel plasmodesmal machinery that has been leveraged to gain further understanding of plasmodesmata function in lateral root formation (Benitez-Alfonso *et al.*, 2013) and immune signalling (Faulkner *et al.*, 2013). Proteomic analyses also generate an expanding ‘parts list’ that allows us to ask whether recurrent protein classes are found at plasmodesmata in multiple plant tissues and species, and thus define a core protein complement of plasmodesmata (Kirk *et al.*, 2022). However, sampling across differentiated tissues and taxonomic groups is hitherto poor, limiting the scope of such an approach. As plasmodesmata are understood to be a feature conserved across land plants (Brunkard and Zambryski, 2017), expanding our current knowledge relating to the plasmodesmata of flowering plants to extant species belonging to different taxonomic groups would give greater insight into core and conserved plasmodesmal components.

The bryophytes are a group of plants sister to the vascular plants (tracheophytes), with these clades diverging soon [~445 million years ago (Mya)] after the conquest of the land by the green kingdom (~490 Mya) (Morris *et al.*, 2018). Electron microscopy has revealed that plasmodesmata across the tissues of bryophytes share architectural features, such as the outer plasma membrane lining and a central desmotubule (comprised of ER), with flowering plants (Ligrone and Duckett, 1994; Cook *et al.*, 1997; Ligrone *et al.*, 2000). These observations suggest that plasmodesmata are a trait present in the ancestor of all land plants and that elements of their structure observed across diverse extant species are essential to their function, being conserved or repeatedly recruited to plasmodesmata. Other than a limited analysis of the proteins present in plasmodesmata of the giant-celled green alga *Chara corallina* (Faulkner *et al.*, 2005), molecular details about the composition of plasmodesmata outside the flowering plants are lacking, leaving questions of the molecular conservation of plasmodesmata unanswered.

A comparison of extant traits and molecular constituents between living ancestors would provide a powerful entry point towards establishing which plasmodesmal components are core, and which are derived. In recent years, extant species from Bryophyta such as *Marchantia polymorpha* and *Physcomitrium* (formerly *Physcomitrella*) *patens* have grown to be important models for plant research (Rensing *et al.*, 2020; Naramoto *et al.*, 2022). We took advantage of recent developments in methods for extracting plasmodesmata from differentiated green tissues to phylogenetically expand information of the molecular composition of plasmodesmata, generating new plasmodesmal proteomes from differentiated tissue of *Arabidopsis thaliana* and *P. patens.* Leveraging these and existing proteomes, we performed a comparative phylogenetic analysis, exploiting a Bayesian approach of repeated identification indicating conserved plasmodesmal association, thereby increasing the power in analysis of recalcitrant plasmodesmal samples. Thus, we identified core plasmodesmal proteins in consistently identified protein orthogroups validating members of β-1,3-glucanase, C2 lipid-binding protein, and tetraspanin families as conserved, core plasmodesmal proteins. Our approach, and new resources, has revealed essential features of plasmodesmata, with the potential to define basic rules and requirements of symplastic cell–cell communication in the multicellular green lineage.

## Materials and methods

### Plant material and growth conditions

For plasmodesmal extraction, *A. thaliana* Col-0 plants were grown on soil in short-day conditions (10 h light/14 h dark) at 22 °C. Leaves were harvested 5 weeks after germination. For stable transformation, *A. thaliana* plants were grown in long-day conditions (16 h light/8 h dark). *Physcomitrium* (*Physcomitrella*) *patens* tissues for generating plasmodesmal fractions were grown on BCD-AT medium in long-day conditions (16 h light/8 h dark) at 25 °C. Protonemal tissue was grown on top of nitrocellulose membrane for 1 week, whereas gametophore tissue was grown directly on the medium for 4 weeks. Routine *P. patens* culture for generating and maintaining transformants was performed under continuous light at 25 °C on BCD-AT medium. *Nicotiana benthamiana* plants were grown on soil with 16 h light/8 h dark at 23 °C.

### Plasmodesmal purification

Plasmodesmata were extracted from expanded rosette leaves of 5-week-old Arabidopsis plants and a mix of *P. patens* protonemal and gametophore tissue. To fully homogenize differentiated tissue, we extracted plasmodesmata according to Cheval *et al.* (2020), with the key difference in approach from that in Faulkner and Bayer (2017) being the homogenization method and the use of Triton X-100 to disrupt chloroplasts. First, frozen mature tissue was ground into a powder in liquid nitrogen and suspended with extraction buffer [EB: 50 mM Tris–HCl pH 7.5, 150 mM NaCl, 1× cOmplete™ ULTRA protease inhibitors (Sigma), 1 mM phenylmethylsulfonyl fluoride (PMSF), 1% (w/v) polyvinylpyrrolidone (PVP)-40 kDa (Sigma)], and ultrasonicated for 1 min in six 10 s pulses with a 5 s pause between each pulse (Soniprep 150 Plus, MSE). The sample was passed twice through a high-pressure homogenizer (EmulsiFlex™-B15, Avestin) at 80 psi. Triton X-100 (10% v/v) was added dropwise to the resultant homogenate to a final concentration of 0.5% (v/v) to disrupt residual chloroplasts, and cell walls were collected by centrifugation at 400 *g*. The cell wall pellet was washed three times (four for *P. patens* samples) with EB (15 ml) and centrifuged at 400 *g*. We validated the method for *P. patens* by calcofluor staining of cell walls at the different stages of fractionation (Supplementary Fig. S1), showing that the size of cell wall fragments generated by this approach is similar to those derived from *A. thaliana* suspension cells (30–100 µm) (Grison *et al.*, 2015).

The cleaned cell wall pellet was incubated in an equal volume of cellulase buffer [CB: 20 mM MES-KOH pH 5.5, 100 mM NaCl, 2% w/v Cellulase R-10 (Yakult Pharmaceutical Co., Ltd, Japan), 1× cOmplete™ ULTRA protease inhibitors (Sigma), 1 mM PMSF] for 1 h at 37 °C, 200 rpm. Undigested cell wall was removed by centrifugation at 5000 *g*, and the supernatant was collected as the plasmodesmal membrane-containing fraction. The cell wall pellet was washed again with CB to extract residual plasmodesmal membranes and the soluble ­fraction was ­ultracentrifuged at 135 000 *g* for 1 h. The membrane pellet was resuspended in 50 mM Tris–HCl pH 7.5, 150 mM NaCl, 5 mM DTT, 1×cOmplete™ ULTRA EDTA-free protease inhibitors (Sigma), 1 mM PMSF, 0.2% (v/v) IPEGAL®CA-630 (Sigma).

### Mass spectrometry

Plasmodesmal samples were run 5 mm into a 1.5 mm thick 10% polyacrylamide Tris resolving gel (containing 0.1% SDS) without a stacking gel, in a glycine 0.1% SDS running buffer. The gel was washed in dH_2_O and then the band was excised. The bands were washed four times in 20% acetonitrile at 40 °C for 15 min to remove detergents, and then stored at 4 °C with 100 µl of dH_2_O.

MS analysis was performed by the Cambridge Centre of Proteomics. 1D gel bands were cut into 1 mm^2^ pieces, destained, reduced (DTT) and alkylated (iodoacetamide), and subjected to enzymatic digestion with trypsin overnight at 37 °C. Digested peptides were analysed by LC-MS/MS with a Dionex Ultimate 3000 RSLC nanoUPLC (Thermo Fisher Scientific Inc., Waltham, MA, USA) system and a Q Exactive Orbitrap mass spectrometer (Thermo Fisher Scientific Inc.). Separation of peptides was performed by reverse-phase chromatography at a flow rate of 300 nl min^–1^ and a Thermo Scientific reverse-phase nano-Easy-spray column (Thermo Scientific PepMap C18, 2 µm particle size, 100 Å pore size, 75 µm i.d.×50 cm length). Peptides were loaded onto a pre-column (Thermo Scientific PepMap 100 C18, 5 µm particle size, 100 Å pore size, 300 µm i.d.×5 mm length) from the Ultimate 3000 autosampler with 0.1% formic acid for 3 min at a flow rate of 15 µl min^–1^. After this period, the column valve was switched to allow elution of peptides from the pre-column onto the analytical column. Solvent A was water+0.1% formic acid and solvent B was 80% acetonitrile, 20% water+0.1% formic acid. The linear gradient employed was 2–40% B in 90 min (the total run time including column washing and re-equilibration was 120 min).

The LC eluant was sprayed into the mass spectrometer by means of an Easy-spray source (Thermo Fisher Scientific Inc.). All *m/z* values of eluting ions were measured in an Orbitrap mass analyser, set at a resolution of 70 000, and scanned between *m/z* 380 and 1500. Data-dependent scans (Top 20) were employed to automatically isolate and generate fragment ions by higher energy collisional dissociation [HCD; normalized collision energy (NCE): 25%] in the HCD collision cell, and measurement of the resulting fragment ions was performed in the Orbitrap analyser, set at a resolution of 17 500. Singly charged ions and ions with unassigned charge states were excluded from being selected for MS/MS, and a dynamic exclusion of 20 s was employed.

Post-run, all MS/MS data were converted to mgf files and the files were then submitted to the Mascot search algorithm (Matrix Science, London UK, version 2.6.0) and searched against the Cambridge Centre of Proteomics database, including common contaminant sequences containing non-specific proteins such as keratins and trypsin. Variable modifications of oxidation (M) and deamidation (NQ) were applied, as well as a fixed modification of carbamidomethyl (C). The peptide and fragment mass tolerances were set to 20 ppm and 0.1 Da, respectively. A significance threshold value of *P*<0.05 and a peptide cut-off score of 20 were also applied. All data (DAT files) were then imported into the Scaffold program (Version 4.10.0, Proteome Software Inc., Portland, OR, USA). Proteins were classed as positively identified when the peptide and protein identification probability thresholds were >95% (Leijon *et al.*, 2018) and proteins were identified in at least two replicates.

### Gene Ontology analysis

Gene Ontology (GO) was used to test gene lists for cellular localization enrichment (Ashburner *et al.*, 2000). A cellular localization GO term over-representation test was performed, using the Panther database (release 1 July 2022) (Thomas *et al.*, 2003; Mi *et al.*, 2019) and GO database (released 13 October 2022) with a Fisher’s exact test and false discovery rate (FDR) reported. *Physcomitrium patens* genes were annotated bioinformatically using phylogenetic backpropagation of GO terms via the Panther database (Gaudet *et al.*, 2011). Graphs were drawn using ggplot2 in R (v4.0.0) (Wickham, 2016).

### Bioinformatic analysis

HMMER v3.3 (hmmer.org) was used for sequence similarity searches (Eddy, 1998). The *P. patens* plasmodesmal proteome was downloaded as peptide sequences from UniProt and used as the reference database for a ‘phmmer’ search against which the *A. thaliana* UniProt proteome was run (UP000006548, accessed 24 April 2020) (Cheng *et al.*, 2017). Protein matches were filtered at either E <1 × 10^–100^ or E <1 × 10^–50^ as stated in the text.

Orthofinder (v2.2.6) was used to create *de novo* orthogroups (Emms and Kelly, 2015, 2019). Plasmodesmal proteome protein sequences were downloaded using UniProt, TAIR (Araport11), and Phytozome v12.1 (*Populus trichocarpa* v3.1). Orthofinder was run on these sequences with default settings.

### Phylogenetic analysis

A peptide sequence was downloaded from UniProt for each protein within an orthogroup. The protein FASTA sequences were aligned with Clustal Omega (v1.2.4; Sievers *et al.*, 2011) to build a consensus sequence. The consensus sequence, in Stockholm format, was used as the basis for a hmmsearch (EBI, HmmerWeb version 2.41.1; Potter *et al.*, 2018). A search was conducted against the EMBL Reference Proteomes database restricted to *A. thaliana* (taxon id: 3702), *P. patens* (taxon id: 3218), and *P. trichocarpa* (taxon id: 3694) species sequences with the sequence E-value cut-off 1 × 10^–100^, unless otherwise stated. Protein sequences were manually deduplicated for each gene.

The FASTA sequences for all identified homologues, from the hmmsearch, in all three species were downloaded and a bootstrapped non-rooted phylogenetic tree was generated using the ‘standard_trimmed_phyml_bootstrap’ ete workflow (v3.1.1; Huerta-Cepas *et al.*, 2016). In this workflow, sequences are aligned with Clustal Omega, trimmed with TrimAI (Capella-Gutiérrez *et al.*, 2009), and a phylogeny determined with 100 bootstraps using PhyML (Guindon *et al.*, 2010). Trees were drawn using ggtree in R (v4.0.0) (Yu *et al.*, 2017).

Molecular phylogeny for *P. patens* plasmodesmata-associated protein families (Supplementary Fig. S2) was determined using the maximum likelihood method (JTT matrix-based model) after sequences were aligned using MUSCLE run on MEGA (v7). A discrete Gamma distribution with five categories was used to model evolutionary rate differences among sites. All positions with <80% site coverage were eliminated.

### Construct generation for protein tagging in moss

For mNeonGreen tagging of moss candidate plasmodesmal proteins, first a mNeonGreen tagging vector was generated. For this the mNeonGreen coding sequence was amplified using primers mNG-HindIII-F and mNG-stop-EcoRI-R (Supplementary Table S1) from pPY22 [Addgene plasmid #137082 (Yi and Goshima, 2020), introducing a GSGGSG-encoding linker before mNeonGreen in the process]. Next, using *Hin*dIII and *Eco*RI restriction sites, the Citrine fluorophore in pCTRN-nptII (Hiwatashi *et al.*, 2008) was exchanged with the amplified mNeonGreen-encoding sequence, resulting in plasmid pmNG-nptII.

Moss mNeonGreen in-locus tagging constructs were assembled using InFusion recombination of PCR-amplified fragments. Four fragments were assembled: a vector backbone sequence amplified from pmNG-nptII using primers pBS-vec-PmeI-F and pBS-vec-PmeI-R; two gDNA-amplified homology arms of ~1 kb in length located upstream and downstream of the intended mNeonGreen integration site; and a mNeonGreen-encoding fragment (Supplementary Fig. S3) which, in the case of C-terminal fusions, was followed by a G418 resistance cassette (both amplified from pmNG-nptII using primers mNG-noStart-F+mNG-noStop-R or Link-mNG-F+Cassette-R, respectively). The resultant plasmids were verified by sequencing and linearized by *Pme*I digestion prior to transformation into *P. patens*.

### Construct generation for *N. benthamiana* and *A. thaliana* expression

For localization analysis of putative plasmodesmal candidates by expression in *N. benthamiana* and *A. thaliana* tissues, binary vectors containing the coding sequence of the protein of interest fused to a fluorescent protein were generated. Typically, the coding sequence of a gene of interest was synthesized (Genewiz, China) as a Golden Gate L0 module in a pUC57 backbone, except for moss tetraspanin PpTET6 (A9TQE7; Pp3c4_3550V3), moss β-1,3-glucanase PpGHL17_18 (A0A2K1J8R8; Pp3c16_15860V3), and Arabidopsis β-1,3-glucanase AtBG_PPAP (Q9FHX5; AT5G42100) (see below). For synthesis, internal *Bsa*I and *Bpi*I restriction sites were removed via silent mutation, and appropriate 4 bp overhangs were added to enable Golden Gate cloning. Via a *Bsa*I-mediated level 1 Golden Gate reaction, coding sequences were linked to an enhanced green fluorescent protein (eGFP)-, mCherry-, or mRuby3-encoding fragment with a 35S or ACT2 promoter and terminator regions placed upstream and downstream, respectively. Coding sequences for PpTET6 and PpGHL17_18 were amplified from moss cDNA and assembled into a Golden Gate level 0 acceptor plasmid, removing internal *Bsa*I and *Bpi*I sites in the process. For the β-1,3-glucanase, during fragment assembly an mNeonGreen-coding fragment was fused in-frame after the sequence coding for the catalytic domain. The tagging constructs for AtBG_PPAP and the N-terminal fusions of Q4A3V3 and AtGELP91 were generated by inserting mCitrine downstream of their predicted signal peptides and assembling the fusion in a Level 1 binary vector with a 35S promoter and either a 35S or heat shock protein terminator.

### Plant transformation

*Arabidopsis thaliana* was transformed by floral dip (Clough and Bent, 1998). *Nicotiana benthamiana* was transformed by co-infiltration of *Agrobacterium tumefaciens* [GV3101 (pMP90)] strains harbouring either a binary plasmid coding for *in planta* expression of the transgene of interest or the p19 silencing suppressor. Leaves were imaged 2 d post-infiltration.

*Physcomitrium patens* was transformed using polyethylene glycol (PEG)-mediated protoplast transformation (Nishiyama *et al.*, 2000). For constructs without a resistance cassette (i.e. those used for N-terminal or internal tagging of the protein of interest), the plasmid p35S-LoxP-HygR (pTN186; Genbank AB542059.1) was co-transformed in a 1:1 ratio such that a first selection step on hygromycin B-containing medium could be performed. Transformants with the correct single integration of the mNeonGreen expression constructs were identified using PCR.

### Confocal imaging

Leaf tissue from *N. benthamiana* and *A. thaliana* was cut into 1 cm^2^ samples and mounted adaxially. Samples were imaged on a ZEISS LSM800 confocal microscope with a ×63/1.2 water immersion objective lens (C-APOCHROMAT ×63/1.2 water). GFP and mCitrine were excited at 488 nm with an argon laser and collected at 500–545 nm. mRuby was excited at 561 nm with a DPSS laser and collected at 590– 620 nm. Aniline blue [0.1% (w/v) in 1× phosphate-buffered saline (PBS) pH 7.4] was infiltrated adaxially and excited at 405 nm with a UV laser and collected at 430–470 nm. Wall fractions were stained with 0.01% Calcofluor white M2R (F3543, Sigma) and imaged by confocal microscopy with a ×20 objective (PLAN APOCHROMAT NA 0.8). Calcofluor white was excited at 405 nm with a UV laser and collected at 430–470 nm.

Moss protonemal cells were observed using a 39 mm diameter glass bottom dish, prepared with solidified BCD medium and grown for 4–6 d in a thin layer of the same medium except solidified with 0.7% (w/v) low melting agarose. For all moss fluorescence microscopy experiments, the second and third caulonemal cells relative to the tip of a protonemal filament were used. Imaging of endogenous moss proteins tagged with mNeonGreen was performed on a spinning disc confocal microscope consisting of a Nikon Ti-eclipse body equipped with a Yokogawa CSU-X1 spinning disc head and ×100 Plan Apo VC objective (NA 1.40). Image digitization was performed with a Photometrics Prime 95B cMOS camera with a ×1.2 post-magnification fitted in front of the camera. Typical exposures used were 500–3000 ms. For excitation of mNeonGreen, a 491 nm laser line was used and emitted light was filtered using a 527/60 bandpass emission filter. All microscope components were operated by MetaMorph software. Co-localization of aniline blue-stained callose deposits with mNeonGreen-tagged proteins of interest was performed on a Leica Stellaris 5 confocal microscope. Aniline blue prepared as a 1.6% (w/v) stock solution in 0.1 M phosphate buffer (pH 8.5) according to Muller *et al.* (2022) was diluted in water to a final concentration of 0.02% in water and then added to the imaging dishes for 48 h prior to observation [except for co-localization of β-1,3-glucanase PpGHL17_18 (A0A2K1J8R8) where a 2 h incubation was used]. Cells were imaged using a ×100 HC plan apo objective (NA 1.40). Excitation of aniline blue was done using a 405 nm solid state laser and emitted light was collected between 420 nm and 490 nm on a HyD detector with the pinhole set to 0.6 Airy units. Excitation of mNeonGreen was done using 505 nm light obtained from a pulsed white light laser, and emitted light was collected between 515 nm and 560 nm on a HyD detector, with the pinhole aperture set to 1 Airy unit. Frames of the two different probes were collected successively and a line-averaging factor of 8 was used.

## Results

### Generation of a plasmodesmal proteome from mature Arabidopsis leaves

There are currently two published plasmodesmata proteomes of *A. thaliana* (Fernandez-Calvino *et al.*, 2011; Brault *et al.*, 2019) that use suspension culture cells as biological material, as well as another from *P. trichocarpa* cell suspension cultures (Leijon *et al.*, 2018). To define a novel *A. thaliana* plasmodesmata proteome that represented differentiated tissue, we extracted plasmodesmata from expanded leaves (assumed to be mature leaves, Kalve *et al.*, 2014) of 5-week-old plants and characterized the proteome by MS. Proteins were considered positively identified in the same manner as in Leijon *et al.* (2018): if the protein (95% certainty; Searle, 2010) was represented in at least two of the three samples by at least one peptide (95% certainty; Keller *et al.*, 2002). With these thresholds, 238 proteins were identified in the fraction (Supplementary Table S2).

**Fig. 1.**
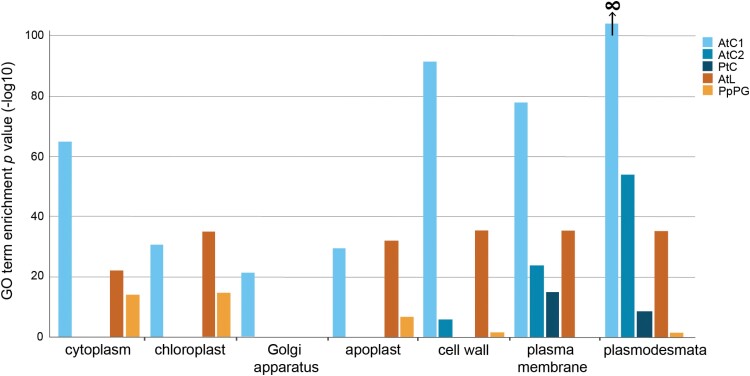
The Arabidopsis plasmodesmal fraction derived from expanded rosette leaves and the moss plasmodesmal fraction derived from protonema and gametophore tissue are enriched in plasmodesmal proteins. *P*-values for cell compartment GO term enrichment of plasmodesmal proteomes from cell suspension cultures (AtC1, AtC2, and PtC), expanded rosette leaves (AtL), and moss protonema and gametophore tissue (PpPG).

To assess if the mature leaf plasmodesmal fraction has sufficient purity to define a plasmodesmal proteome, we assessed whether it showed enrichment for the cellular localization ‘plasmodesma’ GO term. For ease of referencing, hereafter the proteomes are named ‘AtC1’ (Fernandez-Calvino *et al.*, 2011), ‘AtC2’ (Brault *et al.*, 2019), ‘PtC’ (Leijon *et al.*, 2018), and ‘AtL’ (the proteome from mature leaves produced in this study). AtL was benchmarked against AtC2 and PtC for ‘plasmodesmal’ enrichment, noting that >50% of proteins in AtC1 are associated with the ‘plasmodesmata’ GO term, leading to the enrichment *P*-value of close to, and rounded to, zero. It was also benchmarked against AtC1, AtC2, and PtC for the proportion of putative contamination from other subcellular compartments. AtC2, PtC, and AtL were significantly enriched for ‘plasmodesmata’-labelled proteins (Fig. 1). Moreover, all flowering plant proteomes were significantly enriched for ‘cell wall’ and ‘plasma membrane’ proteins, which are both structural components of plasmodesmata. (Brault *et al.*, 2019). The enrichment factor filtering used to define the AtC2 proteome worked extremely well, with other likely contaminant categories (e.g. ‘Golgi apparatus’ or ‘chloroplast’) not over-represented, unlike the unfiltered proteomes. However, the similarity between the representation of proteins in non-plasmodesmal cell components in AtC1 and AtL suggests that the latter is of comparable quality and defines a list of candidate plasmodesmal proteins from Arabidopsis leaves.

### Generation of a plasmodesmal proteome from *P. patens*

In addition to proteomes from cell suspension cultures, a plasmodesmal proteome from *N. benthamiana* leaves (Park *et al.*, 2017) exists, but none is available beyond dicotyledonous flowering plants. To expand the phylogenetic representation of plasmodesmal proteomes, we defined a novel plasmodesmal proteome from the moss *P. patens* (termed ‘PpPG’). We purified cell walls from a mix of protonema and gametophore tissue to produce wall fragments comparable in size with those generated during plasmodesmal fractionation from Arabidopsis cell culture (30–100 µm; Grison *et al.*, 2015; Supplementary Fig. S1). We digested the cellulose in these fragments to release plasmodesmal membranes and analysed the proteins extracted from this fraction by MS. Proteins were identified in the same manner as for the Arabidopsis leaf proteome, generating a list of 215 candidate plasmodesmal proteins (Supplementary Table S3). We confirmed that this extraction protocol works in *P. patens* by checking for enrichment of proteins annotated with the plasmodesmal GO term. A total of 185 (86%) of the UniProt identifiers were mapped to the GO database, with plasmodesma-annotated proteins over-represented (seven proteins, *P*=3.19 × 10^–5^, 0.51 proteins expected) in the *P. patens* plasmodesmal fraction (Fig. 1). This value is reduced compared with the flowering plant plasmodesmal proteomes due to poor annotation of *P. patens* proteins within the ‘plasmodesmata’ GO term via phylogenetic backpropagation of Arabidopsis GO terms (Gaudet *et al.*, 2011). Nonetheless, given the poor backpropagation of GO terms, we concluded that identification of several proteins with a plasmodesmata annotation suggests that the extraction protocol produced a protein fraction that probably contains a representative population of plasmodesmal proteins from *P. patens*.

### Phylogenetic comparison of Arabidopsis, poplar, and moss plasmodesmal proteomes reveals orthogroups containing core proteins

To further characterize and compare the composition of the *P. patens* plasmodesmal proteome, we explored different bioinformatic approaches to find orthologous proteins. First, we used a one-to-one homologue database search approach. Using InParanoid 8.0 (pairwise BLAST, defining orthogroups from an ancestral protein sequence) and MetaPhOrs (defining orthogroups from a meta-analysis of many homologue databases), we converted the *P. patens* protein identifiers to their *A. thaliana* homologue identifier (Supplementary Table S4). Only 62 (InParanoid) and 52 (MetaPhOrs) *P. patens* proteins were matched to Arabidopsis proteins by this approach, but performing a GO term analysis on these two lists of Arabidopsis identifiers revealed enrichment of the plasmodesmata GO term (*P*=7.11 × 10^–16^ and 2.82 × 10^–8^ for InParanoid and MetaPhOrs, respectively). However, the low percentage of *P. patens* protein homologues identified (29% and 24%) by this method is too low to allow for the *P. patens* proteome to offer significant power in a comparative analysis.

Our next analysis involved comparing one with many, instead of relying on databases to convert *P. patens* proteins to *A. thaliana* homologues. To this end, we used HMMER (v3.3, profile hidden Markov models) to find the closest homologue for *P. patens* plasmodesmal proteins in *A. thaliana*. Using two arbitrary thresholds of E <1 × 10^–50^ and E <1 × 10^–100^, HMMER matched 147 (68%) and 80 (37%) *P. patens* proteins to *A. thaliana* proteins, respectively. Even at these conservative values, a HMMER search matched more proteins than database lookup tools. However, one to many mapping makes it difficult to translate the *P. patens* proteome members to specific *A. thaliana* proteins. One approach would be to take the most significant (i.e. most likely) homologue for each protein. However, taking *P. patens* A0A2K1JXU2 (‘X8 domain-containing protein’; Associated locus Pp3c10_5480V3) as an example, there are two almost indistinguishable top hits in *A. thaliana*: O49737 (E=4.2 × 10^–101^) and Q8L837 (E=6.3 × 10^–101^), suggesting that it is likely that the ancestral protein of A0A2K1JXU2 has undergone a duplication event in *A. thaliana* giving two equally likely homologues. In essence, this builds orthogroups restricted to one *P. patens* member.

Another consideration when using HMMER to assign homologues is that to find phylogenetically conserved proteins (i.e. to concurrently compare several lists among several species), one list would have to be chosen as the reference frame. Defining the *P. patens* proteome as the reference list allows the distribution of *P. patens* hits across the Arabidopsis proteomes to be compared, but any nuance from comparison between the *A. thaliana* proteomes is lost. Therefore, we tried a third, many to many approach by forming *de novo* orthogroups using the OrthoFinder software (Emms and Kelly, 2019).

OrthoFinder uses a pairwise BLAST approach to build orthogroups from an input set of protein sequences. We used OrthoFinder (v2.2.6) to define orthogroups (OGs) between five plasmodesmal proteomes: AtC1, AtC2, AtL, PpPG, and PtC. This analysis returned 992 orthogroups, of which 289 had more than one member and 288 contained proteins from multiple proteomes (Supplementary Fig. S4. Two orthogroups had members from all proteomes, and 17 had members from four of the five proteomes (Table 1; Supplementary Table S5). We noted that members of the IMK2 orthogroup (OG18) and OG9 both contain receptor-like kinases belonging to the LRR III group, and that the sole member of the calcium-dependent lipid-binding orthogroup (OG50) identified in the Arabidopsis proteomes shows similarity to members of the C2 lipid-binding orthogroup (OG3, phmmer search E=9.6 × 10^–6^). Therefore, OG18 and OG3 were not considered independently. Further, while OG19, representing DUF26-containing proteins that include the PDLPs, is represented in the proteomes from *P. trichocarpa* and *A. thaliana*, it does not have any *P. patens* homologues (Vaattovaara *et al.*, 2019) and so we excluded it as a candidate core orthogroup. We defined the remaining 16 orthogroups as containing proteins that are ‘phylogenetically conserved plasmodesmal proteins’ (Table 1).

**Table 1.** List of orthogroups identified in at least four of five proteomes

Orthogroup	Protein class	No. of proteomes	No. of proteins	Localized at plasmodesmata
OG0	β-1,3-Glucanase	5	27	Benitez-Alfonso *et al.* (2013); Levy *et al.* (2007)
OG1	Peroxidase	4	22	No
OG3	C2 lipid-binding	4	16	Brault *et al.* (2019)
OG4	SKU5	4	13	No
OG5	GDSL esterase/lipase	4	13	No
OG6	Tetraspanin	4	12	Fernandez-Calvino *et al.* (2011)
OG7	ATP-binding cassette	4	11	No
OG8	Aspartyl protease	4	10	No
OG9	Leucine-rich repeat receptor-like kinase	4	10	Grison *et al.* (2019); Fernandez-Calvino *et al.* (2011)
OG10	Leucine-rich repeat extensin-like	4	10	No
OG13	Histone H2B	4	9	No
OG14	Tubulin beta-7	4	9	Blackman and Overall (1998)
OG16	RNA-binding glycine-rich protein	4	8	No
OG18	Inflorescence meristem receptor-like kinase 2	5	7	No
OG19	DUF26-containing protein	4	7	Thomas *et al.* (2008)
OG28	Eukaryotic translation initiation factor 4A	4	6	No
OG40	Subtilisin-like protease	4	5	No
OG50	Calcium-dependent lipid-binding	4	4	No
OG63	Ribosomal protein	4	4	No

### Moss core orthogroup members are plasmodesmal proteins

Rationalizing that plasmodesmata are defined by specialized membranes, we first considered orthogroups for which the representatives detected in the Arabidopsis proteomes have at least *in silico* support for membrane association [i.e. either predicted transmembrane helices or an omega site for glycosylphosphatidylinositol (GPI) anchor attachment]. This led us to refine our initial OGs of interest to: OG0 (β-1,3-glucanase), OG3 (C2 lipid-binding), OG6 (tetraspanin), OG7 (ATP-binding cassette), and OG9 (LRR RLK III). Proteins from OG0 (Benitez-Alfonso *et al.*, 2013; Levy *et al.*, 2007; Rinne *et al.*, 2011), OG3 (Brault *et al.*, 2019), OG6 (Fernandez-Calvino *et al.*, 2011), and OG9 (Fernandez-Calvino *et al.*, 2011; Grison *et al.*, 2019) have already been validated as plasmodesmata associated in Arabidopsis by live imaging of fluorescent protein fusions. We selected OG0, OG3, OG6, and OG7, and identified *P. patens* homologues, all but one present in the *P. patens* plasmodesmal fraction, and further characterized their *in vivo* localization in the native tissues.

**Fig. 2.**
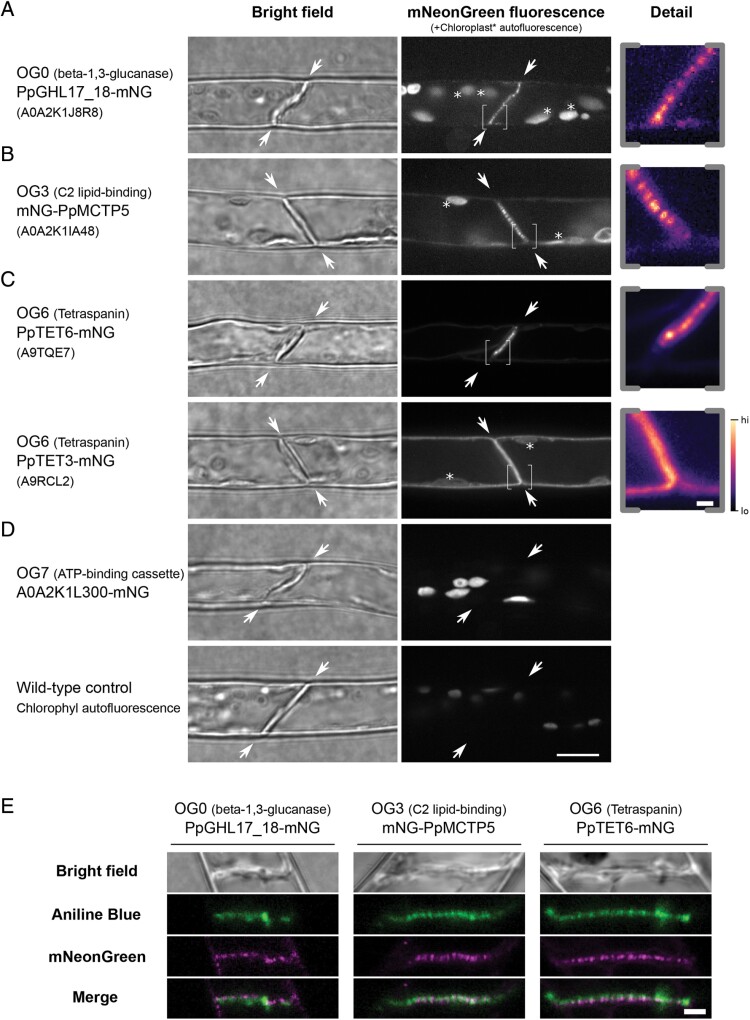
Localization of selected *P. patens* orthogroup members in moss protonemal cells reveals plasmodesmal association. (A–D) Micrographs of moss protonemal cells expressing the indicated protein fused to fluorescent protein mNeonGreen, taken under bright field (left) and fluorescence imaging conditions (right). Proteins belonging to the β-1,3-glucanase (A), C2 lipid-binding (B), tetraspanin (C), and ATP-binding cassette (D) orthogroups were localized. The dividing interface between two neighbouring cells where plasmodesmata are exclusively located in this tissue are highlighted by arrows. When the fusion protein was detected at this location, an expanded view of part of the dividing wall (indicated by brackets) is shown on the right in pseudocolour. Examples of autofluorescent chloroplasts are marked with an asterisk. The A0A2K1L300–mNG fusion protein localized to chloroplasts as levels of chloroplast autofluorescence under the same imaging and display conditions in wild-type tissue were vastly lower (bottom row). Scale bars are 10 µm in overview images, and 1 µm in expanded views. (E) Co-localization of the three mNeonGreen fusion proteins localizing to the cell interface (magenta) with the callose stain aniline blue (green). A single confocal plane is depicted showing co-occurrence of the callose and plasmodesmal protein fusion proteins (merge, bottom row). Scale bar is 1 µm.

For OG0, three *P. patens* β-1,3-glucanases were present in the plasmodesmal fraction (Supplementary Tables S3, S5). We noted that the protein A0A2K1K5L9 (associated locus Pp3c8_940V3) had an incomplete catalytic domain, and therefore disregarded it for further analysis. We selected A0A2K1J8R8 (Pp3c16_15860V3.1, PpGHL17_18, Supplementary Table S6; Supplementary Figs S2, S3), a β-1,3-glucanase with a predicted GPI anchor similar to most known plasmodesmata-associated β-1,3-glucanases (Levy *et al.*, 2007; Benitez-Alfonso *et al.*, 2013; Gaudioso-Pedraza *et al.*, 2018), as a moss representative of OG0. We generated a transgenic *P. patens* line that expresses a fluorescent protein fusion by inserting a mNeonGreen (mNG)-encoding sequence at the native genomic locus downstream of the predicted catalytic domain and before the predicted omega site for GPI anchor attachment (Supplementary Fig. S3). Live imaging of *P. patens* protonema shows that PpGHL17_18-mNG has a punctate localization at the cell junctions (Fig. 2A). Co-localization with aniline blue suggests that this fluorescence pattern is co-incident with staining of plasmodesmal callose (Fig. 2E) and therefore that PpGHL17_18 is a plasmodesmata-associated β-1,3-glucanase.

For OG3, representing the C2 lipid-binding protein family that contains the plasmodesmata-associated MCTPs (Brault *et al.*, 2019), no member was identified in the plasmodesmal fraction from *P. patens* (Supplementary Table S5). Therefore, we selected A0A2K1IA48 (Pp3c27_520V3.1, PpMCTP5, Supplementary Table S6; Supplementary Figs S2, S3) as a candidate *P. patens* plasmodesmal protein as it has the closest homology to Arabidopsis MCTP4 using a phmmer search and is most abundantly expressed in moss tissues (Ortiz-Ramírez *et al.*, 2016; Fernandez-Pozo *et al.*, 2020). We generated a fluorescent protein fusion by homologous recombination, inserting mNeonGreen at the N-terminus of PpMCTP5 and observed a punctate localization restricted to the cell junction (Fig. 2B). Again, aniline blue co-localization confirmed co-incidence of the signal with plasmodesmal callose, validating PpMCTP5 as a plasmodesmal C2 lipid-binding protein from *P. patens* (Fig. 2E). We also noted that PpMCTP5–mNG showed weak ER-associated fluorescence that was enriched at discrete foci at the periphery of the external surface of cells (Supplementary Fig. S5), possibly being points of connection between the ER and the plasma membrane as would be expected for proteins in membrane contact sites.

The tetraspanin group OG6 contained two members identified in the *P. patens* plasmodesmal fraction: A9RCL2 (Pp3c7_23740V3.1, PpTET3, Supplementary Table S6; Supplementary Figs S2, S3) and A9TQE7 (Pp3c4_3550V3.1, PpTET6, Supplementary Table S6, Supplementary Figs S2, S3). mNeonGreen fusions at the C-terminus of these two tetraspanins revealed two different patterns of localization. PpTET6 displayed a punctate pattern of localization at the cell periphery that co-localized with aniline blue staining of plasmodesmal callose (Fig. 2C, E). In contrast, PpTET3 showed even distribution in the periphery of the cell, suggesting that it is not enriched in plasmodesmata but present in the entire plasma membrane (Fig. 2C). Therefore, we validated only PpTET6 as a candidate plasmodesmata-associated protein.

OG7 represents ATP-binding cassette proteins that, in contrast to members from OG0, OG3, and OG6, have not been validated as plasmodesmata-associated proteins in any species. To test whether this group might represent novel core plasmodesmal proteins, we identified A0A2K1L300 in our purified plasmodesmal fraction (Supplementary Table S3) and inserted mNeonGreen by homologous recombination to generate an A0A2K1L300–mNG fusion. Live imaging shows that this protein fusion localizes to chloroplasts, suggesting that it is not enriched in plasmodesmata (Fig. 2D).

### Plasmodesmal association of core orthogroup members is conserved in heterologous species

**Fig. 3.**
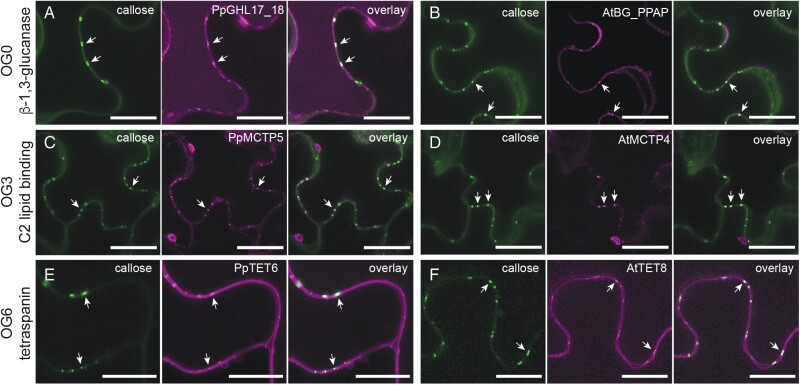
Proteins from OG0, OG3, and OG6 maintain plasmodesmal association in a heterologous species. Confocal micrographs of moss (A, C, E) and Arabidopsis (B, D, F) members of these orthogroups produced in *N. benthamiana* leaf epidermal cells. In each panel, aniline blue-stained plasmodesmal callose is green (left), the candidate–fluorescent protein fusion is magenta (centre), and the overlay is on the right. Members of OG0, representing β-1,3-glucanases, localize to the cell periphery and accumulate at plasmodesmata as indicated by aniline blue co-staining of plasmodesmal callose (arrows). (A) is PpGHL17_18–mNG and accumulates in the vacuole as well as at the cell periphery. (B) is AtBG_PPAP–mCitrine and is detected as diffuse labelling of the cell wall as well as at plasmodesmata. Members of OG3, representing C2 lipid-binding proteins, accumulate at plasmodesmata as indicated by aniline blue co-staining of plasmodesmal callose (arrows). (C) is PpMCTP5–GFP and (D) is AtMCTP4–GFP. Members of OG6, representing tetraspanins, localize to the plasma membrane at the cell periphery and accumulate in plasmodesmata as indicated by aniline blue co-staining of plasmodesmal callose (arrows). (E) is PpTET6–mNG and (F) is AtTET8–mRuby. Scale bars are 20 μm (A, E, F) or 25 μm (B, C, D).

The validation of plasmodesmal association of *P. patens* proteins from orthogroups represented in plasmodesmal proteomes suggests that orthogroup analysis can identify core, conserved plasmodesmal proteins. We reasoned that such core plasmodesmal proteins would be recruited to plasmodesmata in any plant species and, to test this hypothesis, we expressed orthogroup representatives from Arabidopsis and *P. patens* in *N. benthamiana* leaf epidermal cells and used live-cell imaging to determine their association with plasmodesmata. For OG0, we inserted mCitrine downstream of the predicted signal peptide of Q9FHX5 (At5g42100, AtBG_PPAP) and mNeonGreen downstream of the catalytic domain of PpGHL17_18, and expressed the gene fusions transiently in *N. benthamiana* leaves. Both proteins showed punctate distribution across the cell periphery, with foci of fluorescence co-incident with aniline blue-stained plasmodesmal callose (Fig. 3A, B).

Similarly, we generated C-terminal fusions of OG3 members Q9C8H3 (At1g51570, AtMCTP4) and PpMCTP5, and OG6 members Q8S8Q6 (AT2G23810, AtTET8) and PpTET6, with GFP or mRuby and observed punctate localization when expressed in *N. benthamiana* (Fig. 3C–F). These punctae co-localized with aniline blue-stained callose, confirming that these proteins can be recruited to plasmodesmata in a heterologous system. We further confirmed conservation of plasmodesmal association for C2 lipid-binding proteins by stable expression of a GFP fusion of PpMCTP5 in Arabidopsis. Again, this protein fusion localized in punctae at the cell periphery (Supplementary Fig. S6). Thus, β-1,3-glucanases (OG0), C2 lipid-binding proteins (OG3), and tetraspanins (OG6) show characteristics of core plasmodesmal proteins.

### Screening of non-membrane proteins for plasmodesmal association in a heterologous system

**Fig. 4.**
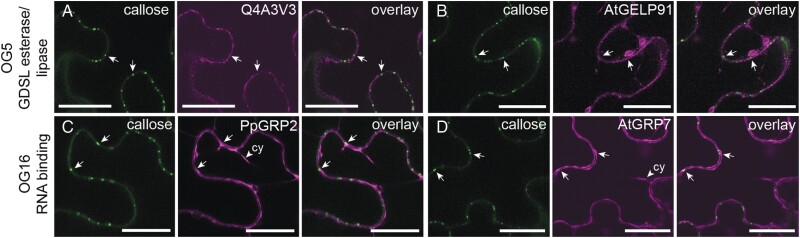
Proteins from OG5 and OG16 do not accumulate in plasmodesmata. Confocal micrographs of *N. benthamiana* leaf epidermal cells producing fusions of moss (A, C) and Arabidopsis (B, D) members of OG5 and OG16. Each panel shows aniline blue-stained callose on the left, the protein–fluorescent protein fusion in the centre, and the overlay of the two images on the right. The positions of two plasmodesmata-associated callose deposits are indicated by arrows in each panel. Members of OG5, containing GDSL esterase/lipases, show an uneven intracellular localization suggestive of a membrane reticulum such as the ER. (A) shows Q4A3V3–GFP and (B) shows AtGELP–GFP (Q9LY84). Members of OG16, containing RNA-binding proteins, show nucleo-cytosolic localization characteristic of soluble proteins. Arrowheads indicate cytoplasmic strands (Cy). (C) shows PpGRP2–mCherry (Q8LPB1) and (D) shows AtGRP7–GFP (Q03250). Scale bars are 25 μm.

Having observed that conserved plasmodesmal proteins maintain their localization in heterologous systems, we used this approach to test the plasmodesmal association of candidates from orthogroups for which members were not predicted to all have a membrane association. We chose Arabidopsis and *P. patens* representatives of OG5 (‘GDSL esterase/lipase’) and OG16 (glycine-rich RNA-binding proteins, GRPs), and screened for plasmodesmal association in *N. benthamiana*. For OG5, we noted that four members were identified in the *P. patens* plasmodesmal fraction. We selected *P. patens* Q4A3V3 (Pp3c18_1550V3.1, the member identified in plasmodesmal fractions with the highest number of peptide hits) and its closest homologue in Arabidopsis Q9LY84 (At5g14450, AtGELP91) for localization analysis. C-terminal protein fusions to GFP showed localization in a cellular reticulum suggestive of the ER (Fig. 4A, B; Supplementary Fig. S7). Co-localization of C-terminal protein fusions with aniline blue-stained plasmodesmal callose showed some reticulum aggregations overlaid with, or adjacent to, plasmodesmata. However, as the ER is continuous with the plasmodesmal desmotubule, and there were many sites where aniline blue signals did not overlay with AtGELP91 or Q4A3V3 fluorescence, we concluded that neither was specifically enriched at plasmodesmata relative to the rest of the ER. To test whether the location of the epitope tag interfered with protein localization, we generated N-terminal protein fusions by inserting mCitrine downstream of the predicted signal peptide of AtGELP91 and Q4A3V3. These localized to intracellular mobile bodies (possibly Golgi bodies) and showed faint diffuse localization at the cell periphery, ­suggesting that the proteins are secreted to the cell wall (Supplementary Fig. S7). While this infers that a C-terminal epitope tag might interfere with the proteins’ exit from the ER, we did not see these OG5 proteins accumulate at plasmodesmata when tagged at either terminus.

For OG16 (GRPs), representatives were identified in both Arabidopsis and *P. patens* plasmodesmal proteomes. We selected Q03250 (At2g21660, AtGRP7) from Arabidopsis as it was represented in two of three Arabidopsis plasmodesmal proteomes, and Q8LPB1 (Pp3c11_19620V3.1, PpGRP2; Nomata *et al.*, 2004) from *P. patens* as it had the highest number of unique peptides identified from our *P. patens* fraction. C-terminal fusions of both Arabidopsis and *P. patens* GRPs to GFP showed a nucleo-cytosolic localization in *N. benthamiana* leaves (Fig. 4C, D; Supplementary Fig. S7). Aniline blue staining of tissue producing AtGRP7–GFP and PpGRP2–mCherry suggests that neither GRP co-localizes with plasmodesmal callose. We further tested the localization of AtGRP7 and PpGRP2 by generating N-terminal protein fusions, but this produced an identical pattern of localization to the C-terminal fusions (Supplementary Fig. S7). Further, stable transformation of Arabidopsis with transgenes that encode C-terminal fusions of AtGRP7 and PpGRP2 produced similar localization patterns (Supplementary Fig. S8). Therefore, neither non-membrane-associated orthogroup showed specific plasmodesmal enrichment and association. Whether this arises because the plasmodesmal fraction of the ER and cytosol cannot be resolved from the cellular pool by light microscopy or because these proteins do not associate with plasmodesmata is unclear.

### Phylogenetic analysis within orthogroups identifies different patterns of evolution of plasmodesmata association

**Fig. 5.**
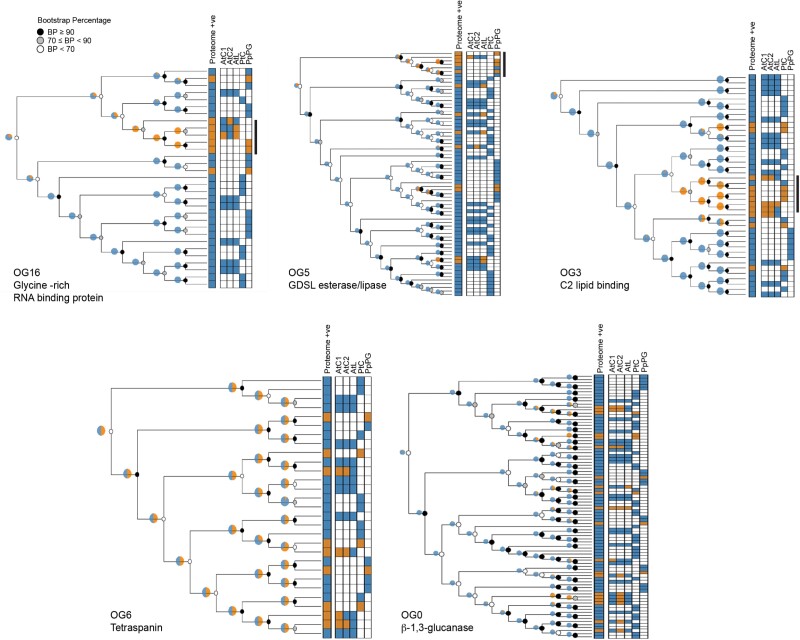
Unrooted cladograms of orthogroup members from *A. thaliana*, *P. trichocarpa*, and *P. patens* as defined by a hmmsearch with a threshold of E <1 × 10^–100^ (OG0/OG3/OG5) or E <1 × 10^–0^ (OG6/OG16). Each tree has a heatmap of proteome matches for each protein in the tree, with orange indicating a proteome hit and blue indicating that the protein was not detected in the relevant proteome(s). For OG3, OG5, and OG16, plasmodesmal association appears to primarily correlate with a single clade within the tree, indicated by the black bar to the right of the proteome heatmap. Pie charts estimate the likely ancestral plasmodesmal localization (orange) by phylogenetic backpropagation. Node support is indicated by greyscale circles.

While live imaging can confirm plasmodesmal association of proteins that accumulate at plasmodesmata such that the fluorescence signal associated with plasmodesmata is greater than or separated from the surrounding pool, the approach is limited when plasmodesmal association is transient and accumulation is not a feature of protein behaviour. We could not confirm plasmodesmal association of OG5 members despite equally strong proteomic support for plasmodesmal association to those of OG6. Therefore, we explored whether protein family phylogenies could identify patterns that indicate a likelihood of conserved plasmodesmal association. We generated unrooted cladograms of the protein families that are represented by orthogroup members from Arabidopsis, poplar, and moss, overlaid the resulting trees with proteomic data, and assessed whether members identified in plasmodesmal fractions were distributed throughout a tree or were clustered in specific clades. OG3, OG5, and OG16, all show plasmodesmal association predominantly in a single branch of the tree (Fig. 5; Supplementary Figs S9–S11), suggesting that plasmodesmal association was gained once during evolution of the protein family. In contrast, plasmodesmal association in OG6 is dispersed across the whole tree, suggesting that each tetraspanin ancestor has an equal likelihood of being associated with plasmodesmata (Fig. 5; Supplementary Fig. S12). Similarly, OG0 (Fig. 5; Supplementary Fig. S13) shows no clear phylogenetic pattern associated with plasmodesmal association. As proteins validated as core plasmodesmal proteins are represented amongst trees that harbour single clades and whole-tree distribution of proteomic hits, this approach offers no further resolution in identifying core plasmodesmal proteins. However, for protein families with plasmodesmal association in specific clades, it offers potential to identify candidate plasmodesmal family members from species for which a proteome has not been generated.

## Discussion

Plasmodesmata are essential features of plant cells, but detailed molecular understanding of their structure and function has long been enigmatic. As membrane-rich structures embedded in the cell wall, they can be described as recalcitrant with respect to biochemical extraction and characterization, and knowledge of their composition has been revealed in a piecemeal fashion despite considerable research efforts. Despite technical challenges, proteomic strategies have underpinned major leaps in understanding of plasmodesmal function, yielding primary knowledge of plasmodesmal responses, as well as formulation of the current model for their core structure being a specialized membrane contact site (Tilsner *et al.*, 2016). Recognizing the gains to be made by better understanding of the protein composition of plasmodesmata in different tissues and species, we used phyloproteomic comparison to define a more detailed atlas of plasmodesmal structure and function.

Defining a proteome is subject to sampling and technical variation that limits the depth of an analysis of samples from a single technical or biological context as subcellular fractionation and MS are inherently noisy techniques (Cargile *et al.*, 2004). The caveat of this is that the most abundant proteins in the preparations will be the most consistently identified, and so some qualitative metric of abundance can be inferred from the repeated presence of a protein. This rationale also applies to a comparison of proteomes of different species in which consistent identification infers conservation and the approach can be used to identify core, essential, and conserved plasmodesmal proteins. Thus, comparative phylogenetic analysis of proteomes from different species gives power to identifying key plasmodesmal components from inherently noisy datasets. With the aim of increasing the analytical power of plasmodesmal proteomics, we generated two new plasmodesmal proteomes from differentiated tissues of Arabidopsis and the moss *P. patens*, and identified protein orthogroups that were represented across samples, hypothesizing that these contain proteins that are core to plasmodesmal structure and/or function.

The ER-derived desmotubule and apoplastic callose have been observed in plasmodesmata across the green lineage (Robards, 1976; Faulkner *et al.*, 2009; Brecknock *et al.*, 2011), suggesting the possibility that these, and other features of plasmodesmata employ families of conserved proteins. We reasoned that core, conserved proteins would associate with plasmodesmata in distantly related plant species, and proteins from β-1,3-glucanase (OG0), C2 lipid-binding protein (OG3), and tetraspanin (OG6) orthogroups demonstrated this behaviour. However, despite evidence of conservation, phylogenetic analysis of the relationships between the moss, poplar, and Arabidopsis protein families from which these orthogroups are derived does not reveal a single pattern of evolution of plasmodesmata association.

Our approach identified and confirmed β-1,3-glucanases and C2 lipid-binding proteins as core and conserved plasmodesmal components. For β-1,3-glucanases this aligns with their characterized role in callose homeostasis at plasmodesmata, with callose deposition detected at plasmodesmata in algae (Faulkner *et al.*, 2009), moss (Fig. 2E; Tomoi *et al.*, 2020; Muller *et al.*, 2022), and flowering plants. Current structural models of plasmodesmata incorporate C2 lipid-binding domain proteins as connectors between the ER and the plasma membrane in specialized membrane contact sites (Brault *et al.*, 2019). Consistent with the conservation of the plasma membrane and desmotubules in plasmodesmata, the conservation of C2 lipid-binding proteins in plasmodesmata suggests that they are a central and core element of plasmodesmata. We observed that the moss C2 lipid-binding protein PpMCTP5 localized at plasmodesmata in moss protonema, but also at other points where the ER sits at the cell periphery (Supplementary Fig. S5) as expected for proteins at membrane contact sites. This further supports the likelihood that there is functional conservation between Arabidopsis and moss C2 lipid-biding proteins and that membrane contact sites are an ancient feature of plasmodesmal structure.

Tetraspanins also showed conserved localization across different species; however, while they have been previously localized to plasmodesmata in Arabidopsis (Fernandez-Calvino *et al.*, 2011), their functional relevance has not yet been established. Tetraspanins are associated with membrane compartmentalization in animals and function in the recruitment and activation of signalling components (Levy and Shoham, 2005; Kummer *et al.*, 2020). For tetraspanins, plasmodesmal association is broadly represented across the cladogram (Fig. 5). As tetraspanins are found across different kingdoms of eukaryotic life, and as our trees are unrooted, it seems unlikely that tetraspanins were an evolutionary advance that specifically catalysed the formation of plasmodesmata. However, these proteins might be associated with the specialization of membrane function associated with the evolution of plasmodesmata. Indeed, as plasmodesmal membranes host localized and specialized signalling cascades, tetraspanins might serve to define the plasmodesmal membrane domain and require further investigation.

With callose deposition central to plasmodesmal function, we were surprised that our analysis did not detect callose synthases. While this might arise from our fractionation methods being suboptimal for their extraction, or from usage of non-quantitative MS methods, we found that if we reduced the stringency of protein identification in both our Arabidopsis leaf and moss plasmodesmal fractions, allowing an identification probability of >50% threshold for peptide and protein identification and a minimum of one sample, we identify an additional 12 orthogroups present in at least four of five proteomes, one of which represents callose synthases (Supplementary Tables S2, S3, S7). This low stringency analysis also reveals orthogroups containing heavy metal-associated isoprenylated proteins (HIPPs), which have been localized to plasmodesmata in Arabidopsis (Guo *et al.*, 2021) and *N. benthamiana* (Cowan *et al.*, 2018), and xyloglucan endotransglucosylase/hydrolase proteins, which have been confirmed as plasmodesmal proteins in a concomitant proteomic study (Gombos *et al.*, 2023). In essence, by requiring the identification of a protein in multiple independent proteomes, we are increasing the *a priori* likelihood of protein identification within a sample. Taking this Bayesian idea, we can reduce the stringent identification criteria of known plasmodesmal proteins, as we are expecting them to appear in the samples. Moreover, proteins which are misidentified would not be classed as ‘core’.

This approach strengthens the confidence in identifying true plasmodesmal proteins by proteomic methods. However, despite their repeated identification in plasmodesmal proteomes, we were unable to resolve any association of proteins in the GRP and GDSL esterase/lipase families with plasmodesmata using confocal microscopy (Fig. 4; Supplementary Fig. S7). The limits of resolution of confocal microscopy suggest that this negative result might not exclude such proteins from having a transient or non-enriched (relative to the rest of the cell) association with plasmodesmata. Future work could use approaches with higher resolution such as immunolocalization by EM or super-resolution light microscopy to determine whether proteins are associated with plasmodesmata.

In addition to the increased power of analysis by comparative analysis, the data contained herein establish new knowledge of moss plasmodesmata. While moss genomes do not encode a family of PDLPs (Vaattovaara *et al.*, 2019), which positively regulate callose deposition (Lee *et al.*, 2011; Caillaud *et al.*, 2014), the detection of stimulus-triggered callose deposition in bryophytes (Kitagawa *et al.*, 2019) suggests that callose regulation of plasmodesmata is an ancestral feature. The absence of PDLPs from moss might indicate the possibility that moss has fewer, or less complex, regulatory processes for callose synthesis.

Cell to cell communication is a central feature of multicellularity. Therefore, a greater understanding of plasmodesmata promises to enhance our knowledge of a whole range of plant processes by resolving which cells and tissues coordinate and communicate to enable organism-level responses. The details of plasmodesmal structure and function are slowly being revealed, and we have demonstrated the benefit of enhancing the knowledge gained from technically difficult proteomic profiling by pooling new and existing information to identify conserved, core plasmodesmal components. Indeed, our approach offers further opportunity to define the core structure of plasmodesmata and expand our understanding across the evolutionary tree to which future efforts can add mechanistic and physiological understanding.

## Supplementary data

The following supplementary data are available at *JXB* online.

Fig. S1. Validation of mature plant tissue fractionation of *P. patens* after successive disruption steps.

Fig. S2. Phylogenetic trees of protein families with plasmodesmata-associated members in *P. patens* inferred using the maximum likelihood method.

Fig. S3. Generation and validation of moss strains expressing candidate plasmodesmal proteins fused to fluorescent protein mNeonGreen.

Fig. S4. Venn diagram representing the overlap between orthogroups identified in each of the proteomes analysed in this study.

Fig. S5. Occurrence of labelled C2 lipid-binding protein-positive punctae at the cell periphery in addition to plasmodesmal association in moss.

Fig. S6. Stable expression of the *P. patens* C2 lipid-binding domain protein PpMCTP5–GFP in Arabidopsis.

Fig. S7. Members of the GDSL esterase/lipase OG5 and the glycine-rich RNA-binding protein OG16 do not associate with plasmodesmata.

Fig. S8. Stable expression of the RNA-binding proteins PpGRP2–GFP and AtGRP7–GFP in Arabidopsis.

Fig. S9. Unrooted cladograms of OG16 (glycine-rich RNA-binding protein) members from *A. thaliana*, *P. trichocarpa*, and *P. patens* with protein Uniprot identifiers listed.

Fig. S10. Unrooted cladograms of OG5 (GDSL esterase/lipase) members from *A. thaliana*, *P. trichocarpa*, and *P. patens* with protein Uniprot identifiers listed.

Fig. S11. Unrooted cladograms of OG3 (C2 lipid-binding) members from *A. thaliana*, *P. trichocarpa*, and *P. patens* with protein Uniprot identifiers listed.

Fig. S12. Unrooted cladograms of OG6 (tetraspanin) members from *A. thaliana*, *P. trichocarpa*, and *P. patens* with protein Uniprot identifiers listed.

Fig. S13. Unrooted cladograms of OG0 (β-1,3-glucanase) members from *A. thaliana*, *P. trichocarpa*, and *P. patens* with protein Uniprot identifiers listed.

Table S1. Primers used in this study.

Table S2. List of proteins identified in *A. thaliana* rosette leaf plasmodesmal fractions by MS, defining AtL.

Table S3. Filtered list of proteins identified in *P. patens* plasmodesmal fractions by MS, defining PpPG.

Table S4. One to one mapping of *P. patens* proteins identified in the plasmodesma-enriched fraction to Arabidopsis homologs using two methods.

Table S5. List of OG members identified in each proteome.

Table S6. Uniprot protein identifiers mapped to genome annotations (EnsemblPlants, Phypa_V3) and proposed names based on phylogenetic relationships.

Table S7. List of orthogroups identified in at least four of five proteomes when the stringency for protein identification in AtL and PpPG is reduced.

erad022_suppl_Supplementary_Figures_S1-S13

erad022_suppl_Supplementary_Table_S1

erad022_suppl_Supplementary_Table_S2

erad022_suppl_Supplementary_Table_S3

erad022_suppl_Supplementary_Table_S4

erad022_suppl_Supplementary_Table_S5

erad022_suppl_Supplementary_Table_S6

erad022_suppl_Supplementary_Table_S7

## Data Availability

MS data generated for this study have been deposited in the ProteomeXchange Consortium via the PRIDE (Perez-Riverol *et al.*, 2022) partner repository with the dataset identifier PXD038964. All other data supporting the findings of this study are available within the paper and within its supplementary data published online.
