# Supplementary material for: Comparative phyloproteomics identifies conserved plasmodesmal proteins
Source: J Exp Bot. 2023 Jan 14;74(6):1821–35. doi: 10.1093/jxb/erad022 (PMC10049917; doi:10.1093/jxb/erad022)
Supplement: erad022_suppl_Supplementary_Figures_S1-S13 [file erad022_suppl_supplementary_figures_s1-s13.pdf]

Supplemental Figures

Figure S1

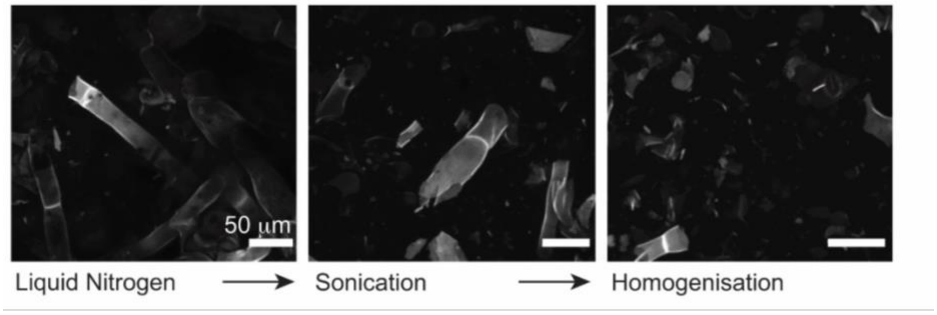

Figure S1: Validation of mature plant tissue fractionation of *P. patens* after successive disruption steps. Tissue fragments stained by Calcofluor White observed by confocal microscopy. Scalebar indicates 50 µm.

Figure S2

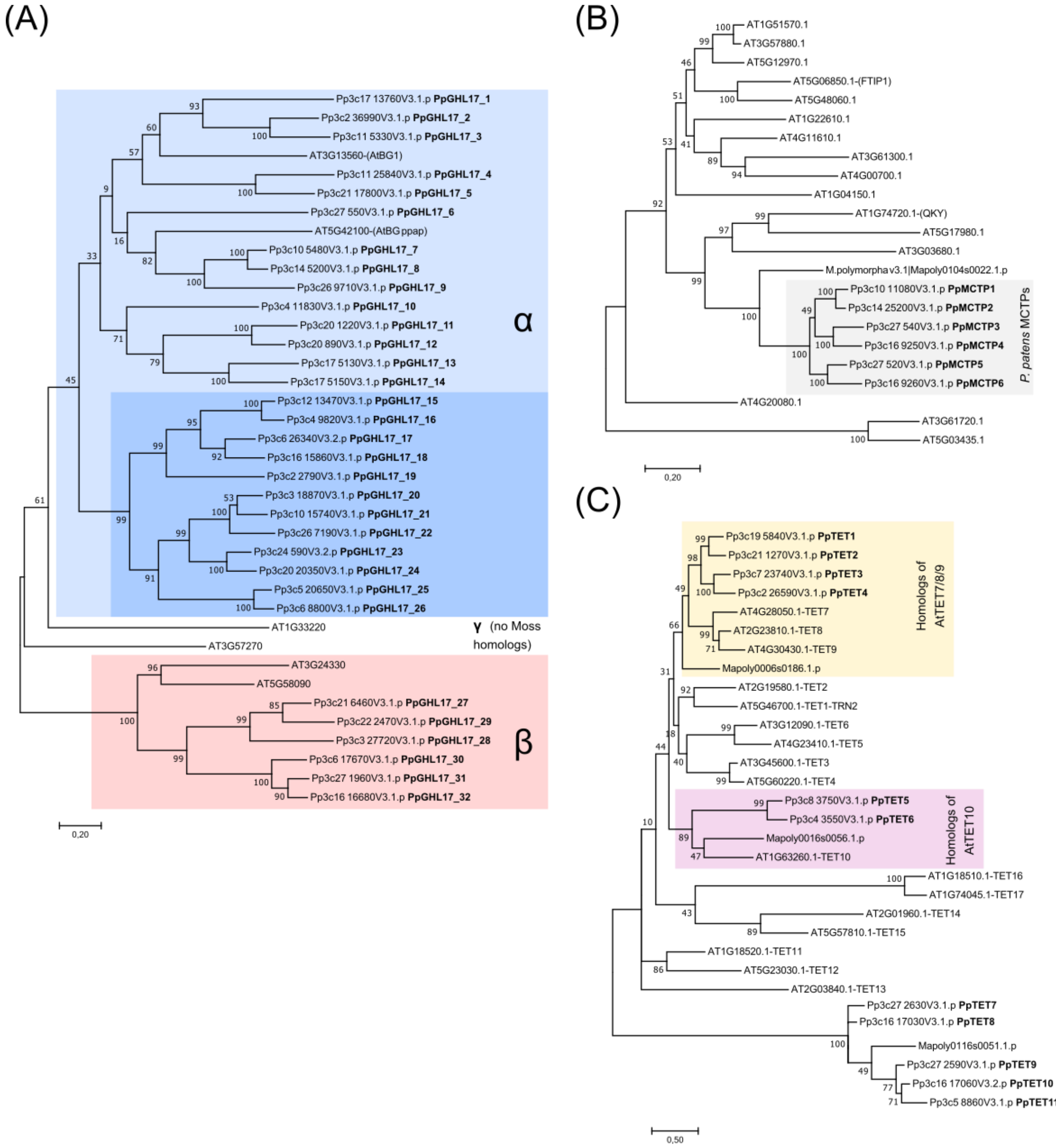

Figure S2: Phylogenetic trees of protein families with PD-associated members in *P. patens* inferred using the maximum likelihood method and names assigned based on the phylogenetic position in bold. Selected proteins from *A. thaliana* and *Marchantia polymorpha* were included as reference. Bootstrap values (derived from 500 replicates) indicate the percentage of trees in which the associated taxa clustered together. Horizontal branch lengths represent the number of substitutions per site. (A) Phylogeny of *P. patens* GHL17 / β-1,3-glucanases, with 2 Arabidopsis representatives each from the α, β and γ clades included for reference. Twenty-six moss members clustered in the α clade (blue), with 12 members comprising a clear subclade (highlighted in darker blue). Six proteins belonged to the β clade (red). (B) Phylogeny of Arabidopsis, *M. polymorpha* and *P. patens* C2 lipid-binding (MCTP) family proteins. All *P. patens* proteins clustered together in a single clade sister to Arabidopsis proteins AT1G74720.1, AT5G17980.1 and AT3G03680.1 (MCTP15/QKY, MCTP16, MCTP14). (C)

Phylogeny of Arabidopsis, *M. polymorpha* and *P. patens* tetraspanin (TET) proteins. Four moss proteins exhibited clustering with AtTET7-9 (highlighted in yellow) and 2 proteins with AtTET10 (purple), with remaining moss proteins clustering outside of the named Arabidopsis tetraspanins.

Figure S3

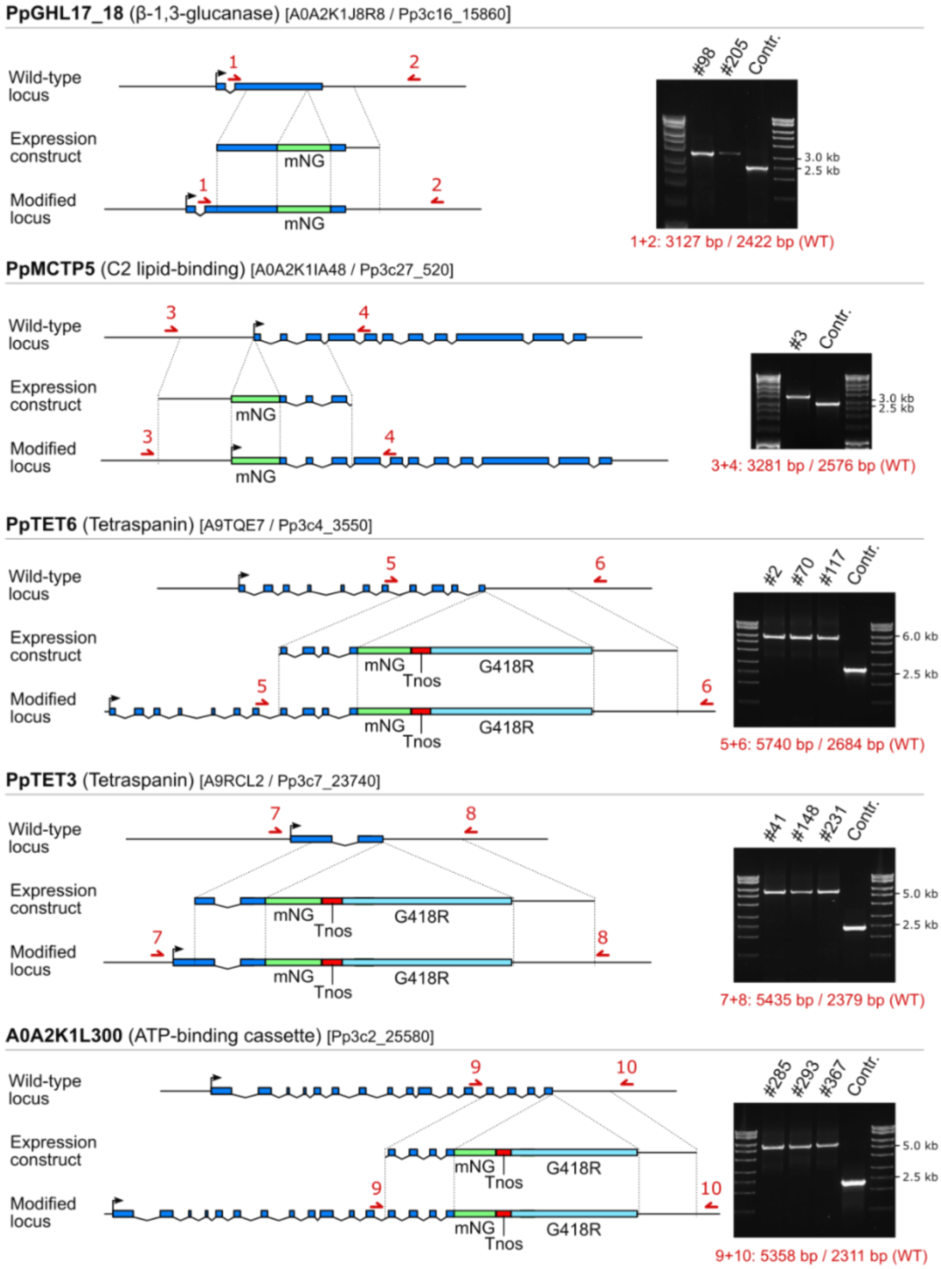

Figure S3: Generation and validation of moss strains expressing candidate plasmodesmal proteins fused to fluorescent protein mNeonGreen. Schematic representations of the genomic locus for each indicated gene with its intron-exon structure (blue boxes) and the constructs used for mNeonGreen tagging via homologous recombination (dashed lines) shown. In tagged lines, a fragment containing the mNeonGreen encoding sequence (green box), and in case of C-terminal tagging also a nopaline synthase terminator (red box) and a cassette conferring G418 resistance (light blue box), is integrated at the start, end or within the coding sequence of the native gene. Red arrows and associated numbers denote primer binding sites used for confirmation of the obtained lines by PCR. The products obtained after PCR reaction and their predicted sizes are given on the right. The numbers of analysed transformants are given above the gel images.

Figure S4

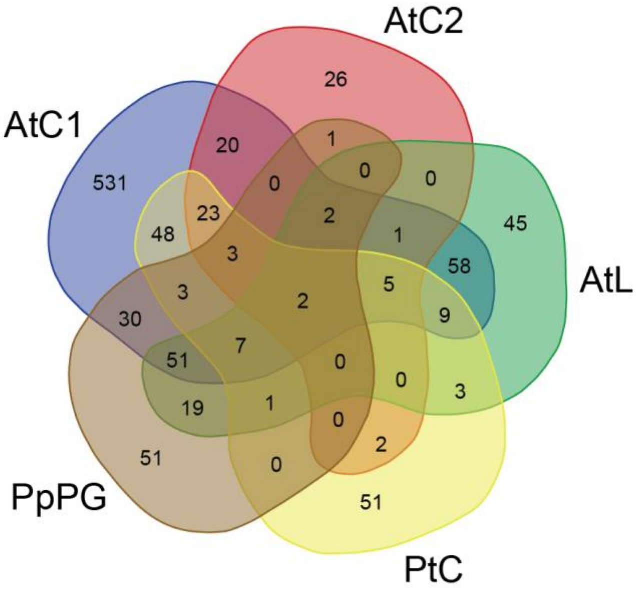

Figure S4: Venn diagram representing the overlap between orthogroups identified in each of the proteomes analysed in this study: AtC1, AtC2, PtC, AtL and PpPG.

Figure S5

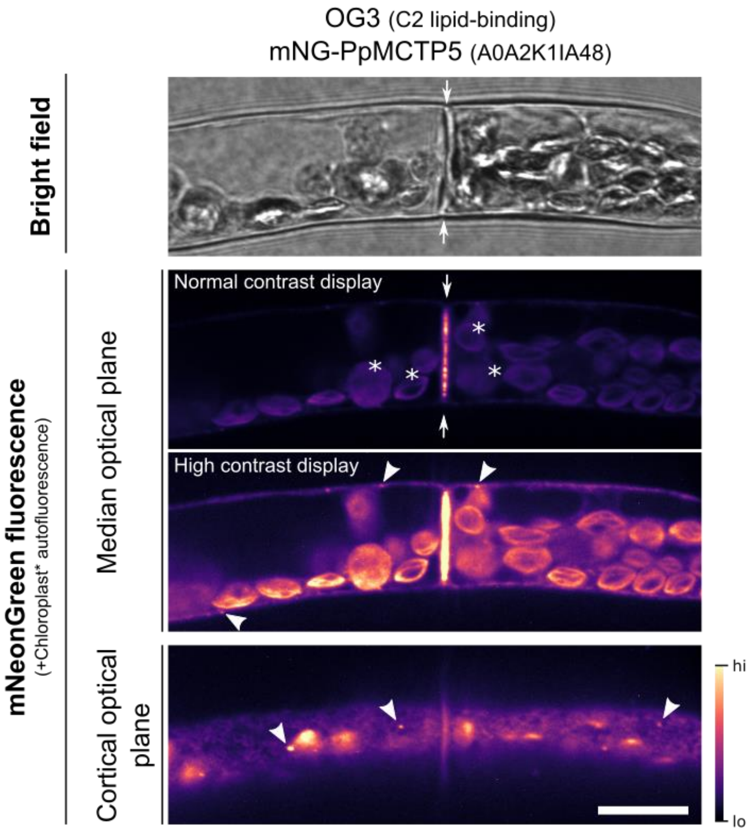

Figure S5: Occurrence of labelled C2 lipid-binding protein-positive punctae at the cell periphery in addition to plasmodesmal association in moss. Two protonemal cells expressing mNeonGreen-PpMCTP5 are shown under bright field (top) and fluorescence imaging conditions. Confocal slices were acquired both in the median plane of the cells and in the cortical plane (bottom). In addition to strong localization to the cell-cell interface (arrows), under high-contrast display settings distinct punctae at the cell periphery were discernible (arrowheads). In the cortical plane the fusion protein also localizes very weakly to reticulate structures, reminiscent of ER. Scale bar indicates 10 µm.

Figure S6

PpMCTP5-GFP

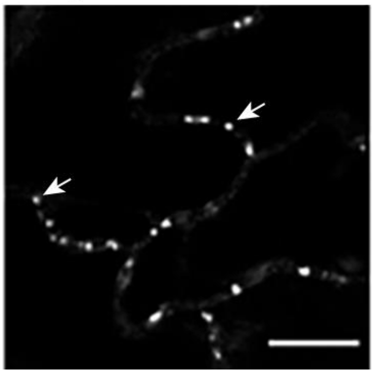

Figure S6: Stable expression of the *P. patens* C2 lipid-binding domain protein PpMCTP5-GFP in Arabidopsis showing accumulation at punctae (arrows) in the cell periphery suggestive of plasmodesmal association. Scale bar is 10 μm.

Figure S7

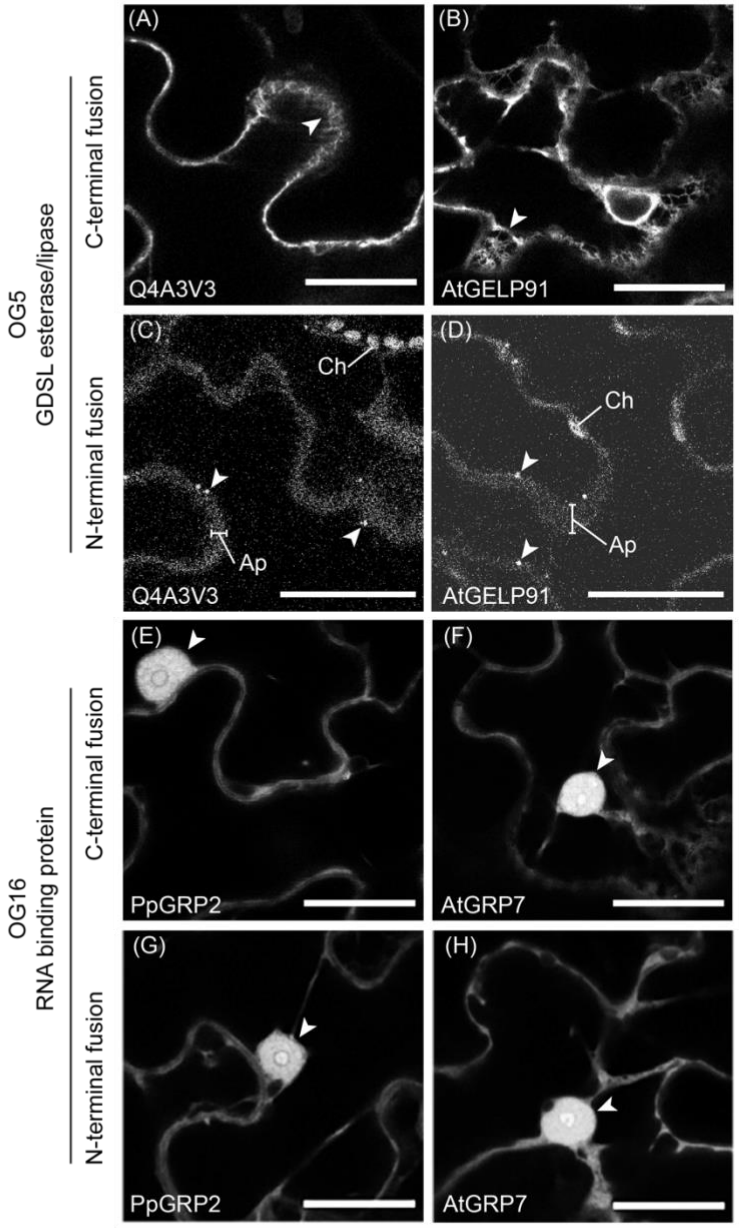

Figure S7: Members of the GDSL esterase/lipase OG5 and the glycine-rich RNA binding protein OG16 do not associate with plasmodesmata. (A-B) Micrographs of single confocal planes show both the *P. patens* (A) and Arabidopsis (B) representatives of OG5 can be seen in a reticulate membrane at the cell surface when C-terminally tagged, (arrowheads) suggesting ER association. (C-D) When the same proteins are N-terminally tagged (downstream of the predicted signal peptide), both the *P. patens* and Arabidopsis OG5 proteins localise to mobile bodies within the cell (arrowheads) and show diffuse localisation in the apoplast (Ap). Fluorescence in the apoplast is very faint when compared to the autofluorescence of the chloroplasts (Ch). (E - H) Micrographs of single confocal planes show both the *P. patens* and Arabidopsis representatives of OG16 are present in the nucleus (arrowheads) and cytosol when C-terminally tagged (A-B) or N-terminally tagged (G-H). Scale bars are 25 μm.

Figure S8

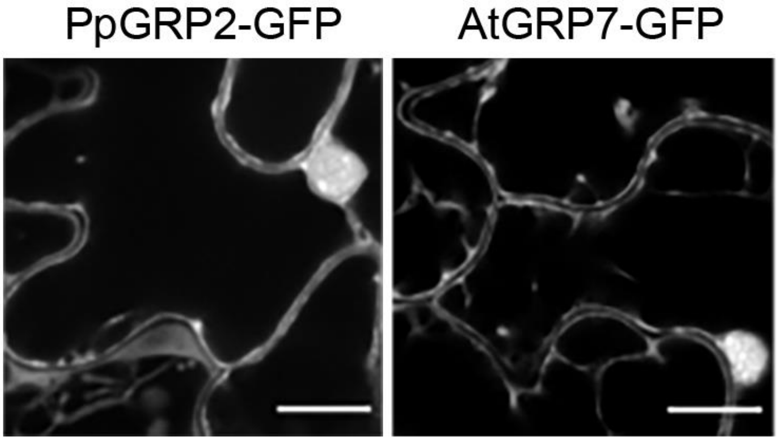

Figure S8: Stable expression of the RNA-binding proteins PpGRP2-GFP (*P. patens*, left) and AtGRP7-GFP (*A. thaliana,* right) in Arabidopsis showing nucleo-cytosolic localisation. Scale bar is 10 μm.

Figure S9

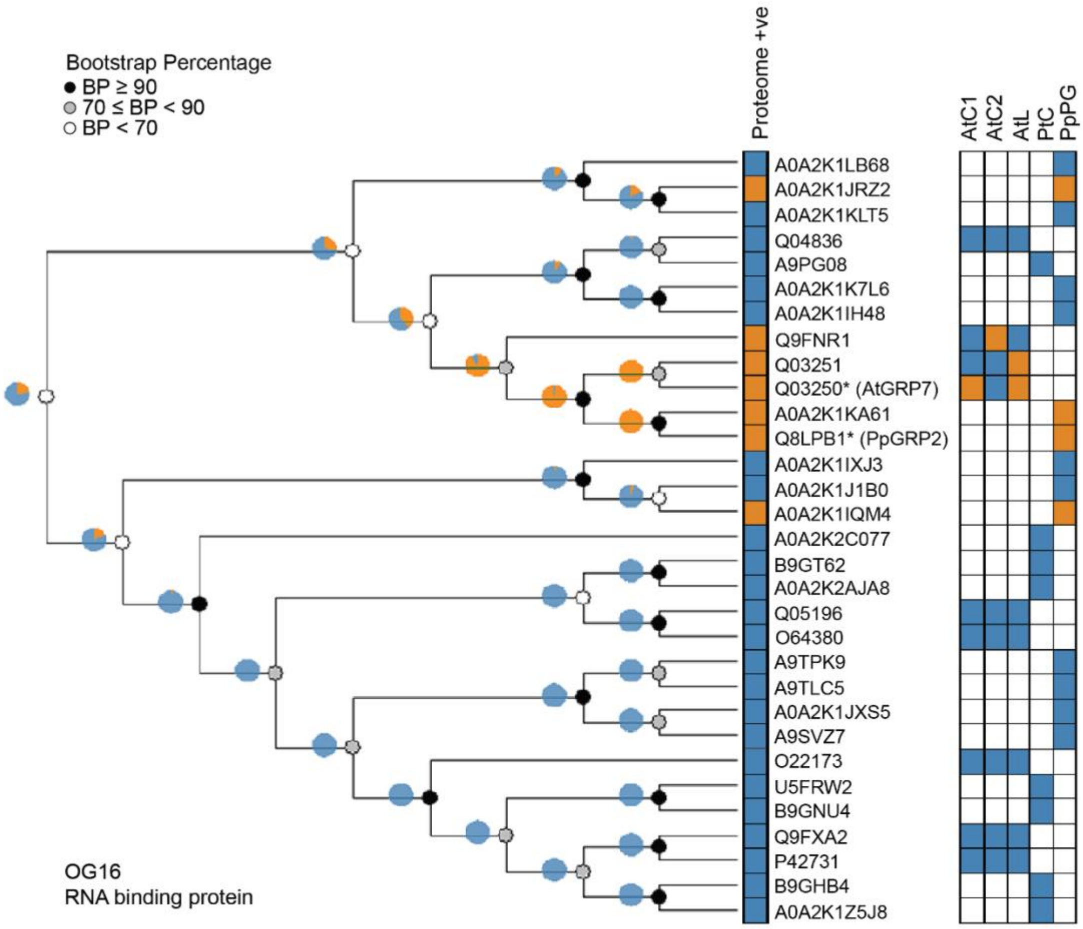

Figure S9: As in Figure 5, unrooted cladograms of OG16 (Glycine-rich RNA binding protein) members from *A. thaliana*, *P. trichocarpa*, and *P. patens* with protein Uniprot identifiers listed. Proteins tested for *in vivo* subcellular localisation are marked with an asterisk. The proteins represented were defined by a hmmsearch with a threshold of E < 1 x 10^{-50}. Each tree has a heatmap of proteome matches for each protein in the tree with orange indicating a proteome hit and blue indicating the protein was not detected in the relevant proteome(s). Pie charts estimate the likely ancestral plasmodesmal localisation (orange) by phylogenetic backpropagation. Node support is indicated by greyscale circles.

Figure S10

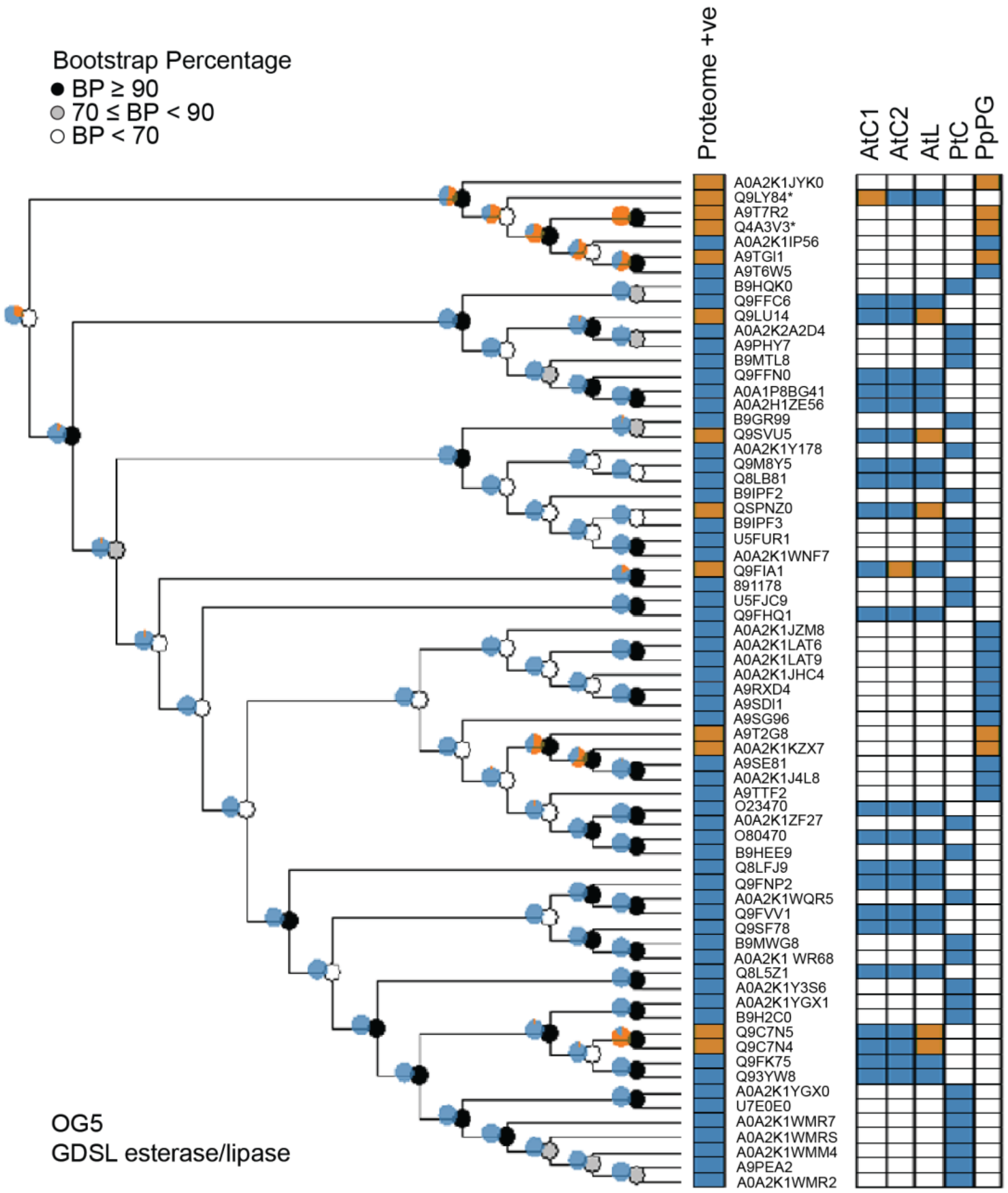

Figure S10: As in Figure 5, unrooted cladograms of OG5 (GDSL esterase/lipase) members from *A. thaliana*, *P. trichocarpa*, and *P. patens* with protein Uniprot identifiers listed. Proteins tested for *in vivo* subcellular localisation are marked with an asterisk. The proteins represented were defined by a hmmsearch with a threshold of E < 1 x 10^{-100}. Each tree has a heatmap of proteome matches for each protein in the tree with orange indicating a proteome hit and blue indicating the protein was not detected in the relevant proteome(s). Pie charts estimate the likely ancestral plasmodesmal localisation (orange) by phylogenetic backpropagation. Node support is indicated by greyscale circles.

Figure S11

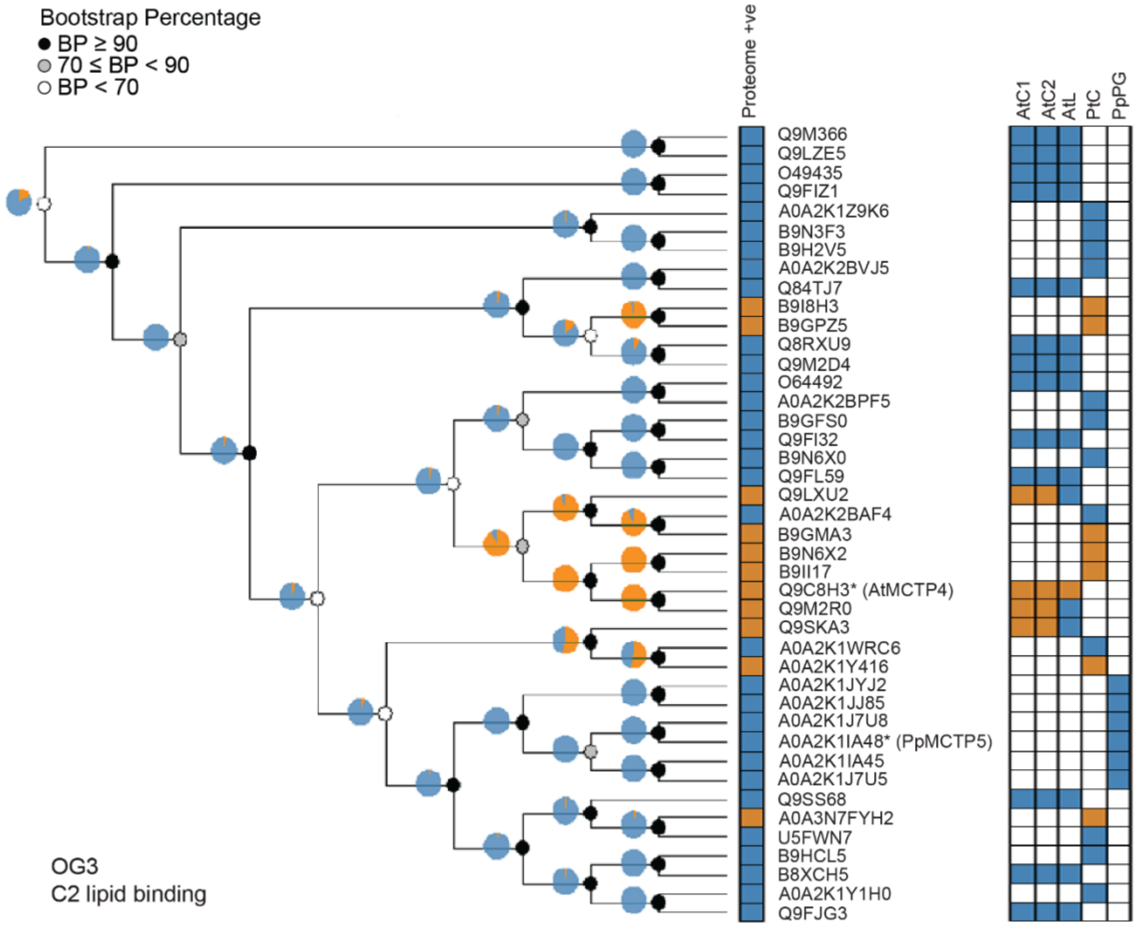

Figure S11: As in Figure 5, unrooted cladograms of OG3 (C2 lipid-binding) members from *A. thaliana*, *P. trichocarpa*, and *P. patens* with protein Uniprot identifiers listed. Proteins tested for *in vivo* subcellular localisation are marked with an asterisk. The proteins represented were defined by a hmmsearch with a threshold of $E < 1 \times 10^{-100}$. Each tree has a heatmap of proteome matches for each protein in the tree with orange indicating a proteome hit and blue indicating the protein was not detected in the relevant proteome(s). Pie charts estimate the likely ancestral plasmodesmal localisation (orange) by phylogenetic backpropagation. Node support is indicated by greyscale circles.

Figure S12

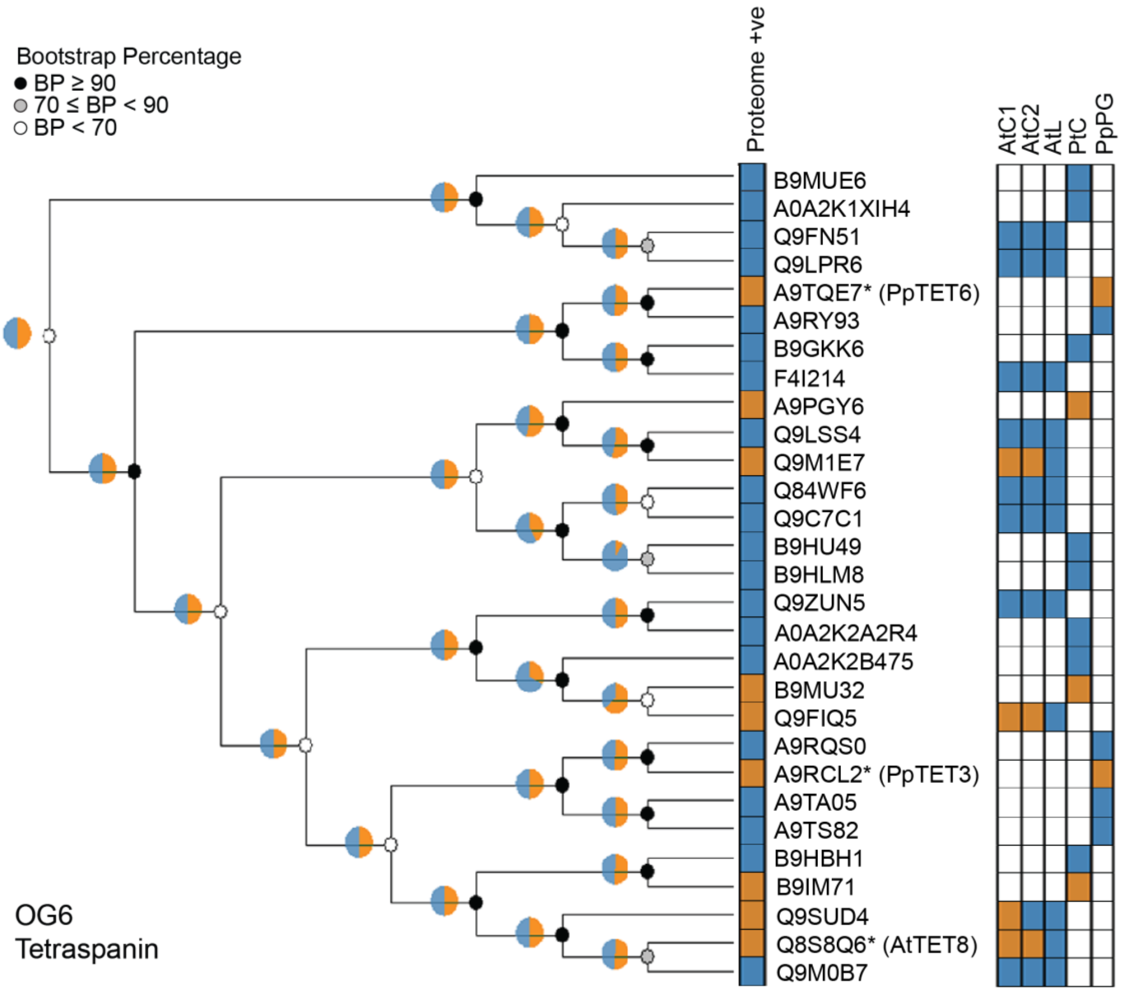

Figure S12: As in Figure 5, unrooted cladograms of OG6 (Tetraspanin) members from *A. thaliana*, *P. trichocarpa*, and *P. patens* with protein Uniprot identifiers listed. Proteins tested for *in vivo* subcellular localisation are marked with an asterisk. The proteins represented were defined by a hmmsearch with a threshold of E < 1 x 10^{-50}. Each tree has a heatmap of proteome matches for each protein in the tree with orange indicating a proteome hit and blue indicating the protein was not detected in the relevant proteome(s). Pie charts estimate the likely ancestral plasmodesmal localisation (orange) by phylogenetic backpropagation. Node support is indicated by greyscale circles.

Figure S13

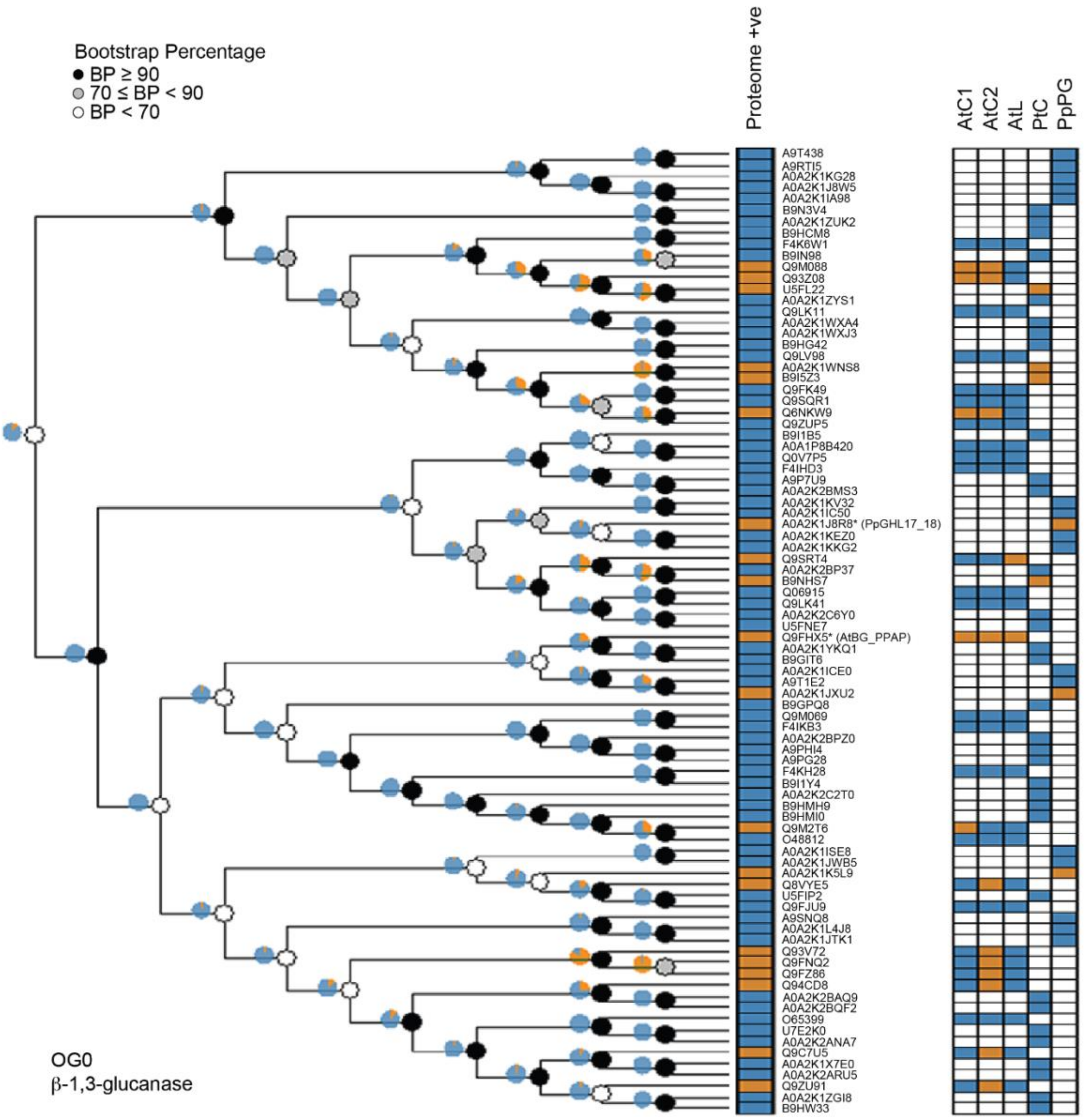

Figure S13: As in Figure 5, unrooted cladograms of OG0 (β-1,3-glucanase) members from *A. thaliana*, *P. trichocarpa*, and *P. patens* with protein Uniprot identifiers listed. Proteins tested for *in vivo* subcellular localisation are marked with an asterisk. The proteins represented were defined by a hmmsearch with a threshold of E < 1 x 10^{-100}. Each tree has a heatmap of proteome matches for each protein in the tree with orange indicating a proteome hit and blue indicating the protein was not detected in the relevant proteome(s). Pie charts estimate the likely ancestral plasmodesmal localisation (orange) by phylogenetic backpropagation. Node support is indicated by greyscale circles.
