# Supplementary material for: Comparative phyloproteomics identifies conserved plasmodesmal proteins
Source: J Exp Bot. 2023 Jan 14;74(6):1821–35. doi: 10.1093/jxb/erad022 (PMC10049917; doi:10.1093/jxb/erad022)
Supplement: erad022_suppl_Supplementary_Table_S7 [file erad022_suppl_supplementary_table_s7.pdf]

Table S7: List of orthogroups identified in at least four of five proteomes when the stringency for protein identification in AtL and PpPG is reduced.

Orthogroup	Protein Class	# Proteomes	# Proteins	In Table 1
OG0	Kinase	5	50	No
OG1	Peroxidase	4	20	Yes
OG2	C2 lipid-binding (MCTP)	4	20	Yes
OG3	Callose synthase	4	15	No
OG4	β-1,3-glucanase	5	14	Yes
OG5	Tubulin beta-7	4	13	Yes
OG6	SKU5	4	13	Yes
OG8	Leucine-rich repeat extensin-like	4	12	Yes
O10	Glycine-rich RNA-binding	4	11	Yes
OG11	Aspartyl protease	4	10	Yes
OG14	RLK	4	10	No
OG15	ATP-binding cassette	5	10	Yes
OG16	Transmembrane protein	5	10	Yes
OG17	Histone 2B	4	9	Yes
OG20	NDR1/HIN1-like protein	5	9	No
OG21	Heavy metal associated isoprenylated plant protein	4	9	No
OG24	DUF26 containing protein	4	8	Yes
OG30	Eukaryotic initiation factor 4A	4	7	Yes
OG32	CSC1-like protein ERD4 (OSCA)	5	7	No
OG35	Xyloglucan endotransglucosylase/ hydrolase	4	7	No
OG40	Subtilisin-like protease	4	6	Yes
OG44	Serine carboxypeptidase-like	4	6	No
OG68	Prohibitin	4	5	No
OG81	Tetraspanin	4	5	Yes
OG88	DUF1191 transmembrane protein	4	4	No
OG102	Ribosomal protein	4	4	Yes
OG103	Molybdopterin biosynthesis	4	4	No
